# Genomic landscape of metastatic colorectal cancer

**DOI:** 10.1038/ncomms6457

**Published:** 2014-11-14

**Authors:** Josien C. Haan, Mariette Labots, Christian Rausch, Miriam Koopman, Jolien Tol, Leonie J. M. Mekenkamp, Mark A. van de Wiel, Danielle Israeli, Hendrik F. van Essen, Nicole C. T. van Grieken, Quirinus J. M. Voorham, Linda J. W. Bosch, Xueping Qu, Omar Kabbarah, Henk M. W. Verheul, Iris D. Nagtegaal, Cornelis J. A. Punt, Bauke Ylstra, Gerrit A. Meijer

**Affiliations:** 1 Department of Pathology, VU University Medical Center, PO Box 7057, 1007 MB Amsterdam, The Netherlands; 2 Department of Medical Oncology, VU University Medical Center, PO Box 7057, 1007 MB Amsterdam, The Netherlands; 3 Department of Medical Oncology, University Medical Center Utrecht, Heidelberglaan 100, 3584 CX Utrecht, The Netherlands; 4 Department of Medical Oncology, Radboud University Medical Centre, Route 452, PO Box 9101, 6500 HB Nijmegen, The Netherlands; 5 Department of Pathology, Radboud University Medical Centre, Huispost 824, PO Box 9101, 6500 HB Nijmegen, The Netherlands; 6 Department of Epidemiology and Biostatistics, VU University Medical Center, PO Box 7057, 1007 MB Amsterdam, The Netherlands; 7 Oncology Biomarker Development, Genentech, Inc., 1 DNA Way, South San Francisco, California 94080 USA; 8 Department of Medical Oncology, Academic Medical Center, PO Box 22660, 1100 DD Amsterdam, The Netherlands

## Abstract

Response to drug therapy in individual colorectal cancer (CRC) patients is associated with tumour biology. Here we describe the genomic landscape of tumour samples of a homogeneous well-annotated series of patients with metastatic CRC (mCRC) of two phase III clinical trials, CAIRO and CAIRO2. DNA copy number aberrations of 349 patients are determined. Within three treatment arms, 194 chromosomal subregions are associated with progression-free survival (PFS; uncorrected single-test *P*-values <0.005). These subregions are filtered for effect on messenger RNA expression, using an independent data set from The Cancer Genome Atlas which returned 171 genes. Three chromosomal regions are associated with a significant difference in PFS between treatment arms with or without irinotecan. One of these regions, 6q16.1–q21, correlates *in vitro* with sensitivity to SN-38, the active metabolite of irinotecan. This genomic landscape of mCRC reveals a number of DNA copy number aberrations associated with response to drug therapy.

## Abstract (web summary)

Response to drug therapy in colorectal cancer (CRC) patients has been associated with tumour heterogeneity. Here the authors analyse DNA copy number aberrations in primary tumours from CRC patients and identify genetic variants that influence drug response.

## Graphical abstract

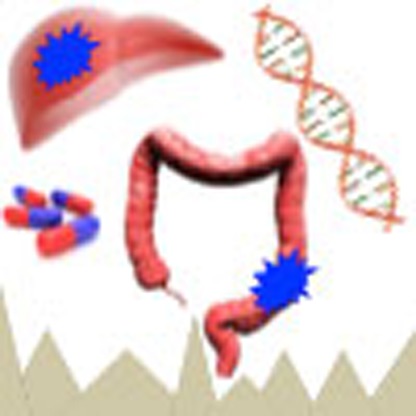

Colorectal cancer (CRC) is the second leading cause of cancer death in the western world with 1.2 million new cases and over 600,000 deaths worldwide in 2008 (ref. 1). CRC results from the accumulation of multiple genetic and epigenetic aberrations^2^, in which patterns are not homogeneous. Based on the paradigm that genotype drives phenotype, in cancer as well as in evolution, inter tumour heterogeneity is thought to be associated to differences in clinical outcome in CRC, in particular to response to systemic treatment. A classic example is failure of anti-epidermal growth factor receptor (*EGFR*) therapy in *RAS* mutated metastatic CRC (mCRC)^3^. However, current systemic treatment for mCRC is still largely based on shot gun approaches (‘one-size-fits-all’), and most patients are treated empirically with fluoropyrimidine-based chemotherapy regimens with or without oxaliplatin or irinotecan and bevacizumab, while the *EGFR*-targeted monoclonal antibodies cetuximab and panitumumab are administered to patients with *RAS* wild-type tumours. To avoid unnecessary toxicity, improve individual patient’s outcomes and constrain healthcare costs, biomarkers predictive of response are needed^4^,^5^. The principle of predictive biomarkers is based on matching the right combination of drugs with particular biological subclasses of CRC.

The biology underlying CRC phenotypes, including response to drug therapy, can be read out at the DNA, RNA and protein level. At this point in time, DNA copy number profiling is the one method that both offers genome-wide coverage and works reliably with formalin-fixed paraffin-embedded (FFPE) tissue samples, which is commonly available, both for use in clinical trials investigating novel (combination) treatment for metastatic disease as well as in routine clinical practice. Moreover, DNA copy number profiling is important to understand the biology of CRC in individual patients, a stepping stone to personalized treatment. For example, DNA copy number aberrations have been found to be predictors of response to the targeted agents trastuzumab, lapatinib and to anthracyclins in breast cancer^6^,^7^,^8^ as well as to carboplatin in ovarian cancer and irinotecan in CRC^9^,^10^. However, apart from HER2 amplification for trastuzumab, clinical validation of these candidate predictive biomarkers is lacking.

In the present study we aim to document the landscape of DNA copy number aberrations in primary tumours of the defined subset of CRC patients who developed metastatic disease and are amenable for systemic treatment. Recent advances in genome technologies have resulted in several series of CRC samples that have been systematically analyzed for genetic aberrations^11^,^12^,^13^,^14^,^15^,^16^. In addition to powerful genomics, homogeneity of phenotypes is also important to derive strong genotype–phenotype associations, which has been a limitation in some series. Samples included in the present study were restricted to those obtained from patients who participated in one of two phase III clinical trials in mCRC, either studying sequential versus combination chemotherapy with capecitabine, irinotecan and oxaliplatin (CAIRO (ref. 17)) or capecitabine, oxaliplatin and bevacizumab with or without cetuximab (CAIRO2 (ref. 18)); *nota bene* cetuximab-treated patients were omitted in the present study given an inferior progression-free survival (PFS) observed with this regimen.

Such data are hypothesis generating in terms of potential biomarkers for prediction of response to the respective drug regimens the patients have been treated with. Moreover it can serve as a catalogue of related amplifications of loci carrying genes against the products of which already drugs exist and have been approved for use. To this end, a large high-quality array comparative genomic hybridization (aCGH) data set of clinically well-annotated CRC specimens was generated using FFPE tumour samples from patients who participated in two phase III clinical trials (CAIRO (ref. 17) and CAIRO2 (ref. 18)).

## Results

### Final data set and descriptives

**Figure 1:**
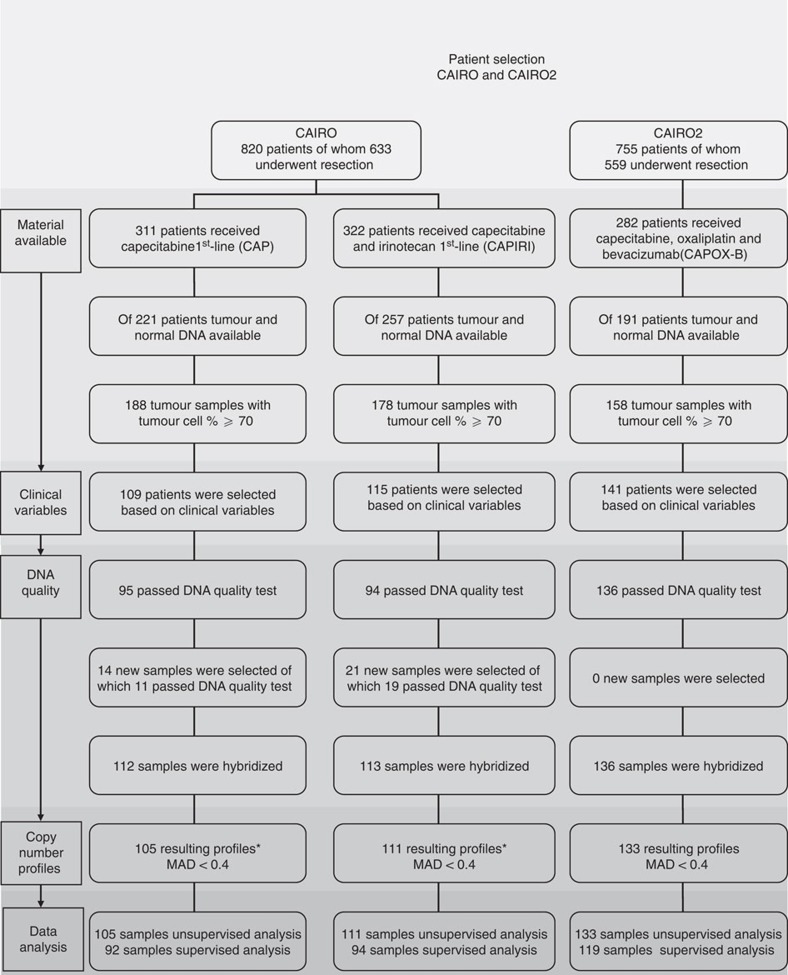
Patient selection and data generation flowchart. The final data set of DNA copy number profiles based on several selection criteria, including clinical variables, DNA quality and quality of the resulting copy number profiles.

After passing all our inclusion criteria the final data set comprised 349 high-quality copy number profiles of 105, 111 and 133 samples of the patient groups; CAIRO arm-A first-line capecitabine (CAP), CAIRO arm-B first-line capecitabine plus irinotecan (CAPIRI) and CAIRO2 arm-A first-line treatment with capecitabine, oxaliplatin and bevacizumab (CAPOX-B), respectively (Fig. 1).

**Figure 2:**
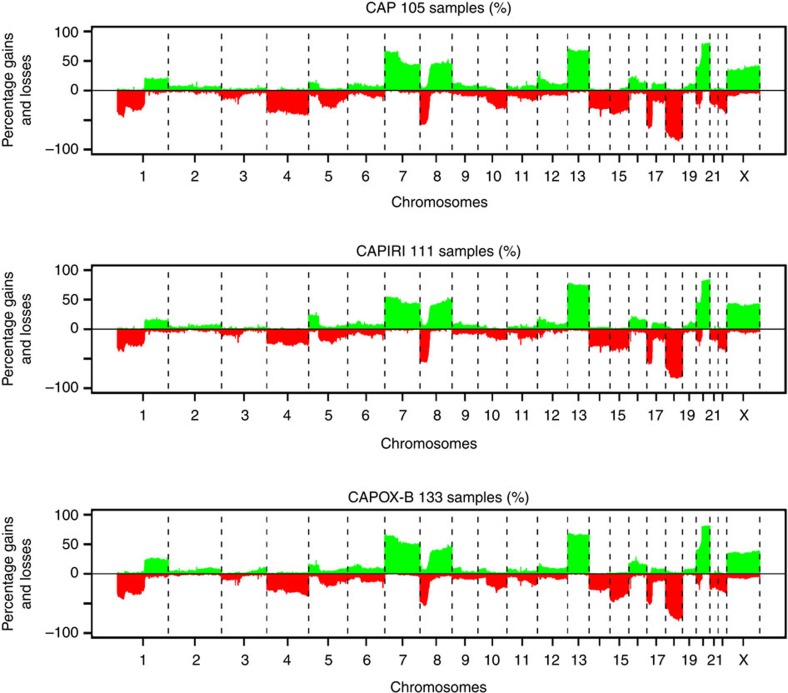
Frequency plots of DNA copy number aberrations for the three mCRC patient groups. Panels from top to bottom CAP, *n*=105 patient samples; CAPIRI, *n*=111 patient samples; CAPOX-B, *n*=133 patient samples. Percentages of DNA copy number aberrations are based on called data. *x*-axis, clones spotted on the array sorted by chromosomal position; vertical-dotted lines, boundaries between chromosomes. *y*-axis, percentage of tumours with gains (positive values) or losses (negative values).

Quality of the obtained DNA copy number profiles was high, both when measured by the median absolute deviation (MAD) quality measure (average 0.17 and s.d. 0.05, range 0.09–0.39) and by visual inspection, shown for three cases in Supplementary Fig. 1. Overall, the frequency distribution of DNA copy number aberrations was similar for all three arms (Fig. 2) and compared with what has been reported previously for CRC^14^,^19^,^20^, with most common gains at chromosomes 7, 8q, 13 and 20 and most common losses at 1p, 4, 8p, 14, 15, 17p and 18. Patient characteristics of the 349 CAIRO and CAIRO2 samples included in the study corresponded well to those of the overall trial population (Supplementary Table 1), implicating that a representative group of patients was profiled. Also the median PFS in the three arms was comparable to PFS in the original studies as a whole (Supplementary Fig. 2) if corrected for patients who underwent resection of the primary tumour. As expected, PFS of the first-line therapy between the three arms differed significantly (Supplementary Fig. 2, *P*-values were 0.0018, <0.001 and 0.004 for CAP versus CAPIRI, CAP versus CAPOX-B and CAPIRI versus CAPOX-B, respectively). This is explained by the different treatment regimens in each study arm, as well as by differences in patient inclusion criteria between CAIRO and CAIRO2.

### Unsupervised analysis of DNA variation

In a first attempt to evaluate the association of genome variation in mCRC to response to first-line systemic treatment, we performed an unsupervised hierarchical cluster analysis including all 349 CRC samples. Unsupervised hierarchical cluster analysis revealed two distinct clusters of 252 and 97 cases, respectively (Supplementary Fig. 3). The *n*=97 cluster contained CRCs with relatively few copy number aberrations. This ‘silent cluster’ also contained the majority of MSI tumours (*n*=19 out of 21, *P*-value *χ*^2^<0.001). However, for none of the three regimens, an association of cluster membership with PFS was found (log-rank *P*-value=0.6, 0.3, 0.5 for CAP, CAPIRI and CAPOX-B, respectively). Even a more detailed subdivision into three clusters (of *n*=77, 175 and 97, respectively) revealed no association (*P*-values are 0.4, 0.2 and 0.3 for CAP, CAPIRI and CAPOX-B, respectively). Since the majority of MSI tumours cluster together and showed infrequent copy number aberrations they were excluded for further analysis.

### Supervised analysis of DNA associations with drug response

**Figure 3:**
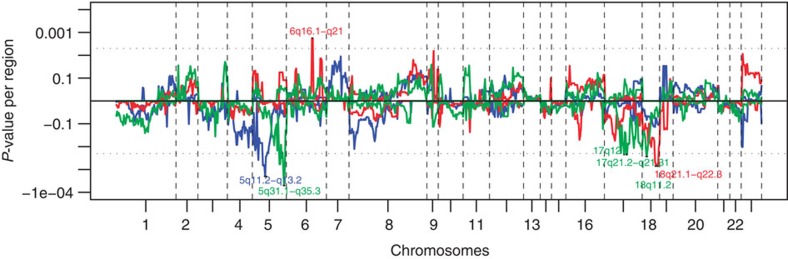
Genome-wide association of PFS with either gain or loss. *x*-axis, clones spotted on the array sorted by chromosomal position; vertical-dotted lines, boundaries between chromosomes. *y*-axis, *P*-values of gains (positive values) or losses (negative values); horizontal-dotted lines show the threshold of significance (log-rank test using 10.000 permutations, *n*=92, *n*=94 and *n*=119 in the patient groups receiving CAP, CAPIRI and CAPOX-B respectively, single-test *P*-value <0.005) and the seven chromosomal bands that cross these lines are noted. The patient group receiving CAP is depicted in blue, CAPIRI in red and CAPOX-B in green.

**Figure 4:**
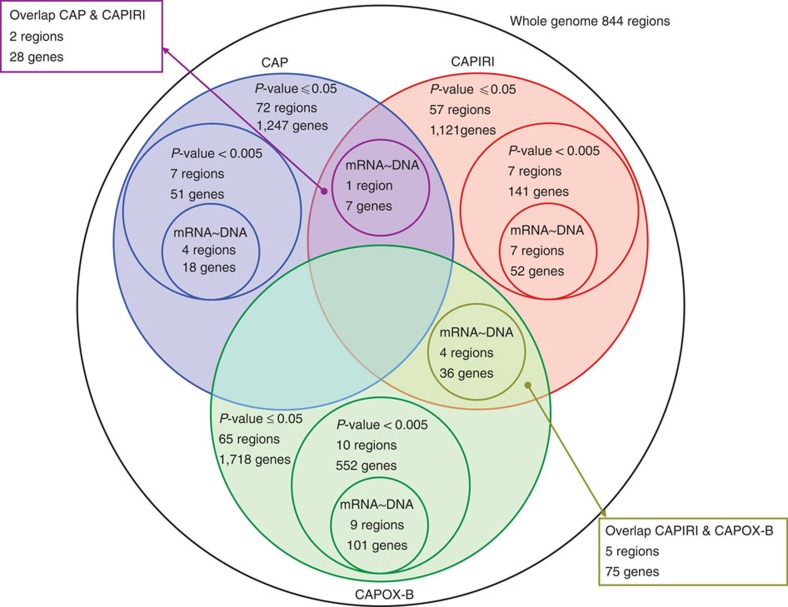
Venn diagram of chromosomal subregions. Chromosomal subregions show significantly different PFS (log-rank test using 10.000 permutations, *P*-value<0.05) for loss versus no loss or gain versus no gain found by supervised analysis. CAP in blue, CAPIRI in red and CAPOX-B in green. Subregions with *P*-value<0.005 and the number of genes located on those regions are shown, as well as the number of genes with correlating DNA copy number and mRNA expression in the TCGA data set.

In the univariate analysis of genome variation associated with response to drug therapy, using the log-rank test, in total 92 and 94 patients receiving CAP and CAPIRI, respectively, and 119 patients receiving CAPOX-B were included (Fig. 1). Out of the 855 variant chromosomal subregions identified in the data pre-processing step, 194 were significantly associated with PFS in at least one of the three treatment regimens at a *P*-value<0.05. On average these subregions covered 3.24 Mb (0.004–23.84 Mb, s.d.=4.28 Mb) (Figs 3 and 4), and in total contain 3,979 messenger RNA (mRNA) coding genes and 144 micro RNAs.

Twenty-four out of the 855 aberrant subregions, contained 744 genes and were associated to PFS with an uncorrected single-test *P*-value of <0.005, shown in Supplementary Table 2. In the CRC The Cancer Genome Atlas (TCGA) data set with paired DNA copy number and mRNA expression data, for 171 of the 744 genes a positive correlation between DNA copy number and mRNA expression level was found (Fig. 4, Supplementary Table 2). Thirty-two out of these 171 genes were found to be mutated in the data set from the ‘The genomic landscapes of human breast and CRCs’ paper^12^ (Supplementary Table 2) and 141 were found to carry mutations in the TCGA data set. In addition, 7 subregions, containing 103 genes, were associated to PFS with an uncorrected single-test *P*-value of <0.05 in at least 2 treatment regimens, shown in Supplementary Table 3. In the TCGA data set, for 43 of these genes a positive correlation between DNA copy number and mRNA expression level was found (Figs 3 and 4, Supplementary Table 3). Five out of these 43 genes were mutated in the data set of ‘The genomic landscapes of human breast and CRCs’ paper^12^ (Supplementary Table 3) and 35 of these genes were mutated in the TCGA data set.

### DNA associations with CAP therapy

For patients treated with CAP as a first-line treatment one locus stood out, related to loss of 5q. This locus (5q11.2–q13.2) concerns seven significant consecutive subregions of 13.5 Mb in total with a significantly shorter PFS (Supplementary Table 2). The most significant of the seven sub-regions (5q12.1–q12.3) was lost in 20 patients for which association reached a *P*-value of <0.001 (median 106 days versus 210 days for loss versus no loss, respectively, Supplementary Fig. 4a). This subregion contains five genes (*SFRS12IP1, SDCCAG10, CENPK, PPWD1* and *SFRS12*) that showed a significant correlation between DNA copy number and mRNA expression in the TCGA data set (Supplementary Table 2). Among patients receiving CAPIRI and CAPOX-B, no association of loss of this 5q region with PFS was seen (Supplementary Fig. 4b and Supplementary Fig. 4c), while the prevalence of this event was similar in all three treatment groups.

### DNA associations with CAPIRI therapy

**Figure 5:**
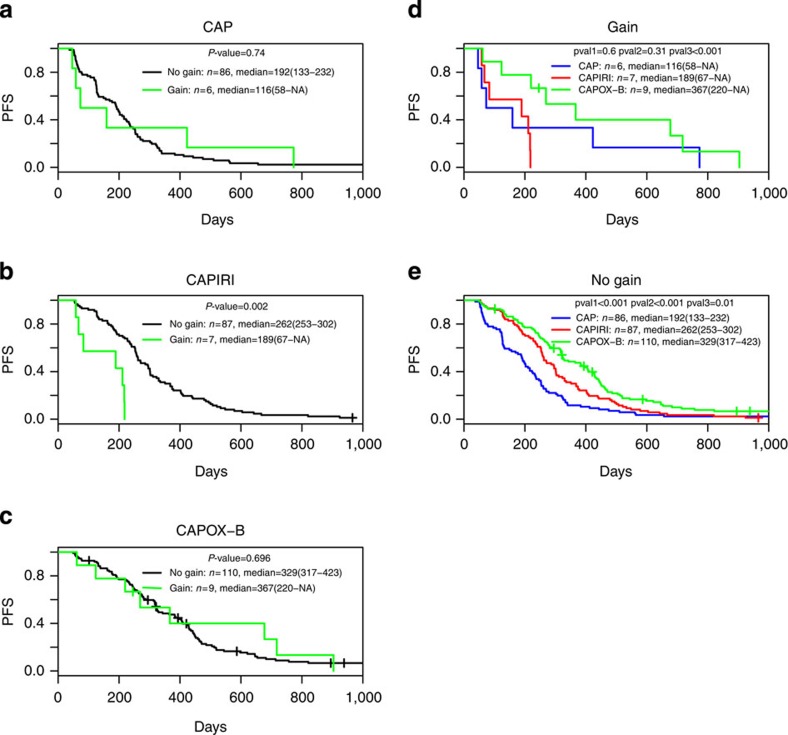
Kaplan–Meier PFS analysis for chromosomal subregion 6q16.1–q16.3. Within patients groups (**a**) CAP; (**b**) CAPIRI; (**c**) CAPOX-B, with gain in green without gain in black. Between patient groups (**d**) gain of 6q16.1–q16.3; (**e**) no gain of 6q16.1–q16.3, with CAP in blue, CAPIRI in red and CAPOX-B in green. Log-rank *P*-values, *P*<0.005 are considered significant; pval1, CAP versus CAPIRI; pval2, CAP versus CAPOX-B; pval3, CAPIRI versus CAPOX-B; *n*, number of patients; median, median survival with confidence limits within brackets and NA, not applicable.

For patients treated with CAPIRI two loci stood out, related to gain of 6q and loss of 18q. The locus on chromosome 6 (6q16.1–q21) concerns two significant consecutive subregions of 14.5 Mb in total with a significantly shorter PFS (Supplementary Table 2). The most significant of the two subregions (6q16.1–q16.3) was gained in 7 patients for which association reached a *P*-value of 0.002 (median 189 days for gain versus 262 days for no gain, Fig. 5b). The total 14.5 Mb region contains 15 genes (*KIAA0776, C6orf66, C6orf167, FBXL4, SFRS18, CCNC, ASCC3, ATG5, QRSL1, 6orf203, PDSS2, LACE1, CD164, SMPD2* and *ZBTB24*) that showed a significant correlation between DNA copy number and mRNA expression in the TCGA data set.

The locus on chromosome 18q (18q21.1–q22.3) concerns five significant consecutive subregions of 24.5 Mb in total with a significantly longer PFS. The most significant of the 5 subregions (18q21.33–q22.3) was lost in 78 patients which association reached a *P*-value of 0.001 (median 270 days for loss versus 181 days for no loss, Supplementary Fig. 5b). The total 24.5 Mb region contains 37 genes (*MYO5B, MBD1, CXXC1, C18orf24, ME2, ELAC1, SMAD4, MEX3C, MBD2, POLI, RAB27B, CCDC68, TXNL1, WDR7, FECH, NARS, ATP8B1, ALPK2, MALT1, SEC11C, LMAN1, PMAIP1, RNF152, PIGN, KIAA1468, TNFRSF11A, ZCCHC2, PHLPP, BCL2, KDSR, VPS4B, SERPINB8, TMX3, RTTN, SOCS6, C18orf55* and *CNDP2*) that showed a significant correlation between DNA copy number and mRNA expression in the TCGA data set. Among patients receiving CAP and CAPOX-B no association of gain of the 6q and loss of the 18q region with PFS was seen (Fig. 5a,c, Supplementary Fig. 5a and Supplementary Fig. 5c), while the prevalence of these events was similar in all three treatment groups.

### DNA associations with CAPOX-B therapy

For patients treated with CAPOX-B three loci stood out, related to loss of 5q, 17q and 18q. The locus on chromosome 5q (5q31.1–q35.4) concerns six significant consecutive subregions of 38.3 Mb in total with a significantly shorter PFS (Supplementary Table 2). The most significant of the seven subregions (5q34) was lost in 15 patients for which association reached a *P*-value of <0.001 (median 196 days for loss versus 390 days for no loss, Supplementary Fig. 6c, Supplementary Table 2). This 66 Kb sub-region contains only one gene (*CCNG1*) that showed a significant correlation between DNA copy number and mRNA expression in the TCGA data set. The total 38.3 Mb region contains 58 genes in total (*CCNG1, BRD8, KIF20A, DC23, ETF1, HSPA9, CTNNA1, SIL1, UBE2D2, C5orf32, PFDN1, ANKHD1, SLC35A4, IK, WDR55, HARS, HARS2, DIAPH1, HDAC3, KIAA0141, RNF14, NDFIP1, LARS, RBM27, TCERG1, PCYOX1L, CSNK1A1, MED7, THG1L, CLINT1, RNF145, UBLCP1, TTC1, SLU7,PTTG1, RARS, CCDC99, NPM1, ERGIC1, C5orf25, HSPC111, HIGD2A, FAF2, GPRIN1, UIMC1, LMAN2, GRK6, DDX41, RMND5B, HNRNPAB, CLK4, ZNF354A, ZNF354B, RUFY1, CANX, MAML1, MAPK9* and *CNOT6*) that showed a significant correlation between DNA copy number and mRNA expression in the TCGA data set.

The locus on 17q (17q12–q21-31) concerns three consecutive subregions of 6.0 Mb in total with a significantly shorter PFS (Supplementary Table 2). The most significant of the three subregions (17q21-q21.31) was lost in 16 patients for which association reached a *P*-value of 0.004 (median 278 for loss versus 389 days for no loss, Supplementary Fig. 7c). The total 6.0 Mb region contains 43 genes (*PSMB3, PIP4K2B, CCDC49, RPL23, LASP1, RPL19, FBXL20, MED1, CRKRS, NEUROD2, STARD3, TOP2A, SMARCE1, TMEM99, KRTAP3-3, KRTAP1-1, EIF1, NT5C3L, KLHL11, ACLY, NKIRAS2, KAT2A, COASY, MLX, EZH1, VPS25, CCDC56, BECN1, PSME3, RUNDC1, RPL27, BRCA1, NBR2, NBR1, DUSP3, TMEM101, LSM12, TMUB2, GPATCH8, CCDC43, EFTUD2, NMT1* and *MAP3K14*) that showed a significant correlation between DNA copy number and mRNA expression in the TCGA data set.

The locus on 18q (18q11.2) concerns only one subregion of 72 kb with a significantly longer PFS (median 390 days for loss versus 256 days for no loss, Supplementary Fig. 7f) and was lost in 78 patients for which association reached a *P*-value of 0.004. However this region does not contain any genes that showed a significant correlation between DNA copy number and mRNA expression in the TCGA data set. However three microRNAs are located in this region (hsa-mir-320c-1, hsa-mir-133a-1 and hsa-mir-1-2). Among patients receiving CAP and CAPIRI, no association of loss of these regions with PFS was seen (Supplementary Figs 6a,b and 7ab,d,e), while the prevalence of this event was similar in all three treatment groups.

### DNA associations with either CAP or CAPIRI therapy

For patients treated with either CAP or CAPIRI as first-line treatment two loci stood out, related to loss (CAP) or gain (CAPIRI) of either 5p14.3-p13.3 or Xp22.33 (Supplementary Table 3). The locus on chromosome 5 concerns one region of 15 Mb, loss of which (*n*=9) was associated with a significantly shorter PFS for CAP (median 106 for loss versus 199 days for no loss). However a gain of this region on chromosome 5 (*n*=23) was associated with a significantly shorter PFS for CAPIRI (median 241 for gain versus 262 days for no gain), suggesting an opposite effect for these two treatments. This region contains seven genes (*RNASEN, C5orf22, GOLPH3, MTMR12, ZFR, SUB1* and *TARS*) that showed a significant correlation between DNA copy number and mRNA expression in the TCGA data set.

The locus on chromosome X concerns one region of 669 Kb, loss of which (*n*=8) was associated with a significantly shorter PFS for CAP (median 66 days versus 197 days for loss and no loss, respectively) and a gain (*n*=31) of which was associated with a significantly longer PFS for CAPIRI (median 335 days versus 254 days for gain and no gain, respectively). This region does not contain any gene that showed a significant correlation between DNA copy number and mRNA expression in the TCGA data set. Furthermore no microRNAs were located in this region.

### DNA associations with either CAPIRI or CAPOX-B therapy

For patients treated with either CAPIRI or CAPOX-B two loci stood out, related to loss of 18p11.32-21 and 18q11.2-12.1 (Supplementary Table 3). The locus on 18p11.32-21 concerns three consecutive subregions of 14 Mb in total, loss of which was associated with a significantly longer PFS for both treatment regimens. This locus contains 35 genes (*USP14, THOC1, C18orf56, TYMS, ENOSF1, YES1, METTL4, NDC80, SMCHD1, EMILIN2, LPIN2, MRCL3, MRLC2, ZFP161, RAB12, KIAA0802, NDUFV2, ANKRD12, TWSG1, RALBP1, PPP4R1, VAPA, NAPG, CHMP1B, MPPE1, IMPA2, TUBB6, AFG3L2, CEP76, PSMG2, PTPN2, SEH1L, CEP192, C18orf19* and *RNMT*) that showed a significant correlation between DNA copy number and mRNA expression in the TCGA data set. Interestingly, one of the genes in this locus is *TYMS*, encoding for the target of capecitabine, thymidylate synthase. However, no significant correlation was found of *TYMS* copy number status and PFS for treatment with CAP. The locus on 18q11.2-12.1 concerns two consecutive subregions of 2 Mb in total, loss of which was associated with a significantly longer PFS for both CAPIRI and CAPOX-B (Supplementary Fig. 8b,c). This locus contains one gene (*LAMA3*) that showed a significant correlation between DNA copy number and mRNA expression in the TCGA data set.

### Across regimen PFS

Associations of PFS across all 305 samples obtained from the three patient groups, CAP, CAPIRI and CAPOX-B, were made and have a prognostic rather than predictive value. An association of PFS with copy number status yielded six chromosomal subregions with a log-rank *P*-value<0.005 (Supplementary Table 4), four of which are located on chromosome 5 and overlap with the subregions found for one arm (Supplementary Table 2). For the other two subregions, loss of 104 kb at 1p31.1 and gain of 3630, kb at chromosome 9q12–q13, significance values of *P*-value 0.005 and 0.004 were observed, whereas no significance was reached within the independent patient groups (Supplementary Fig. 9, shows Kaplan–Meier curves for chromosome 9).

### Between regimen PFS

As outlined above in the sub-section ‘final data set and descriptives’, overall PFS increased for patients treated from CAP to CAPIRI to CAPOX-B. Interestingly, for certain tumour genotypes this situation was different. For patient samples with a loss of 5q12.1–q12.3, the PFS metrics for CAPIRI and CAPOX-B were similar and superior to CAP (Supplementary Fig. 4d), implicating that in this specific subgroup CAPIRI might be a better first-line treatment option compared with CAPOX-B since former therapy is both less toxic and less expensive. For patients without a loss of 5q12.1–q12.3, PFS with CAPOX-B was significantly better compared with the other two regimens (Supplementary Fig. 4e), hence CAPOX-B might be a better treatment option for this subgroup of patients. For patients with a loss of 18q21.33–q22.3, the PFS metrics for CAPIRI was superior to CAP, but without a loss of 18q21.33–q22.3 the PFS metrics were similar. Also for patient samples with a gain of chromosomal subregion 6q16.1–q16.3 PFS metrics were similar for CAPIRI and CAP (Fig. 5d). These examples indicate that adding irinotecan to capecitabine does not have an effect on response in patient groups with either a gain of 6q16.1–q16.3 or without a loss of 18q21.33–q22.3, unlike the subgroup of patients without a gain of 6q16.1–q16.3 or with a loss of 18q21.33–q22.3. Finally, for patients with a loss of subregion 5q34 the PFS was significantly better when treated with CAPIRI, compared with the other two regimens (Supplementary Figs 6d,e).

### *In vitro* validation of DNA associations with drug response

**Figure 6:**
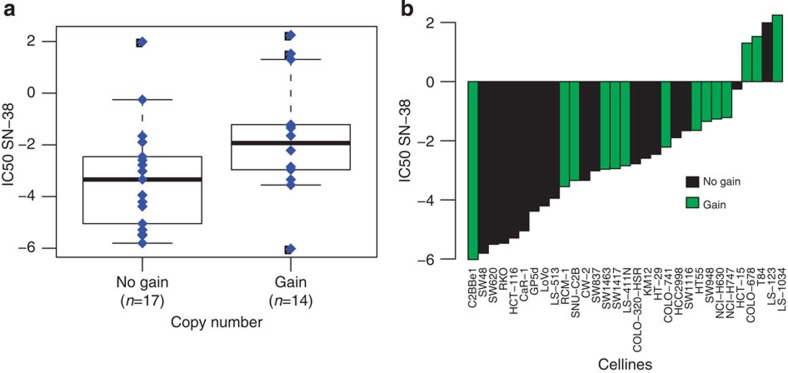
Drug response and chromosome 6q16.1−q21 gain in CRC cell lines. SN-38 drug response for 31 CRC cell lines for chromosomal region 6q16.1–q21. (**a**) Box plot of 6q16.1–q21 copy number, uncorrected Kruskal−Wallis test *P*-value of 0.05; *x*-axis, gain (*n*=14) or no gain (*n*=17); *y*-axis, drug response (IC50 values). The error bars (whiskers) extend to the most extreme data point, but no further than 1.5 times the interquartile range (**b**) Waterfall plot of 6q16.1–q21 copy number (*n*=31); *x*-axis cell-lines ordered from left to right, for low to high IC50 value; *y*-axis, drug response (IC50 values); no gain, black; gain, green.

To evaluate whether, also *in vitro*, presence or absence of the chromosomal regions identified were associated with drug sensitivity, copy number aberrations for cell lines in the Catalogue of Somatic Mutations in Cancer (COSMIC)^21^ were matched with the drug response data (IC50 values) from the Genomics of Drug Sensitivity in Cancer project (GDSC)^22^. Of the four different drugs used in the CAIRO or CAIRO2 clinical trials, response data was available for SN-38, which is the active metabolite of irinotecan, but not for any of the other three drugs used in these regimens. In the between regimen analysis, three (concatenated) chromosomal regions, 6q16.1−q21 gain, 5q11.2−q13.2 loss and 18q21.1−q22.3 loss, associated with a significant difference in PFS between patients treated with irinotecan plus capecitabine and those treated with capecitabine alone (Supplementary Table 2). For the 573 COSMIC cell lines with available GDSC drug response data, a significant association between chromosome 6q16.1−q21 gain and viability under SN-38 treatment was observed (*n*=350 gain, versus *n*=223 no gain, *P*=0.01). Similarly, for the subset of 31 CRC cell lines alone a significant association for gain of 6q16.1−q21 with SN-38 treatment was observed (*n*=31, 14 gain versus 17 no gain, *P*=0.05) (Supplementary Fig. 10a and Fig. 6). *P*-values did not reach significance for either of the other two chromosomal regions that associated with a significant difference in PFS in the patient samples (Supplementary Table 5, Supplementary Figs 10b,c and 11).

### Genes and drug targets in chromosomal amplifications

**Figure 7:**
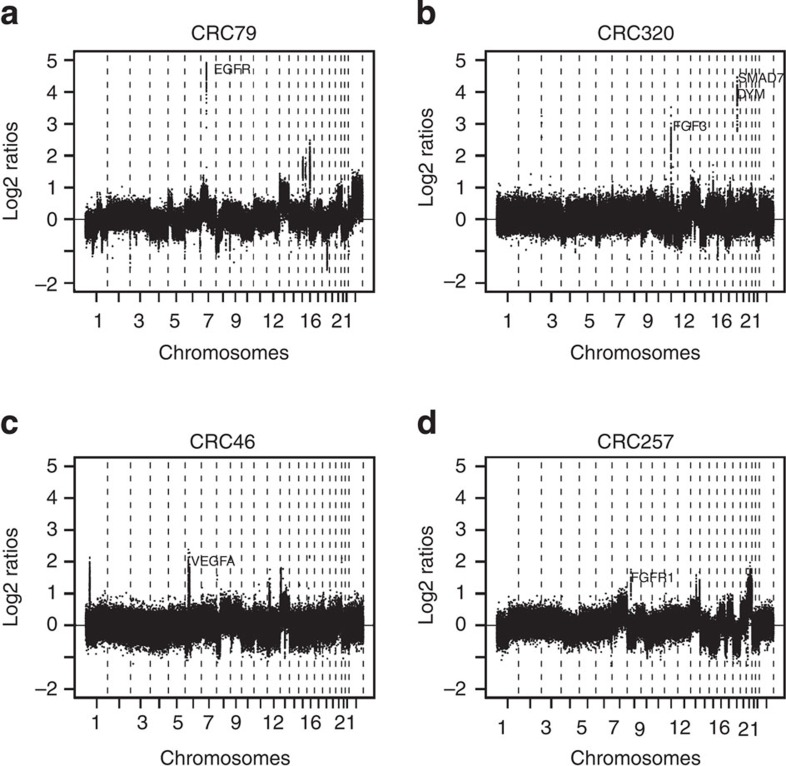
Representative examples of DNA copy number profiles with amplified regions containing known drug targets. Four tumour DNA copy number profiles with amplifications containing the genes (**a**) *EGFR*, (**b**) *FGF3*, (**c**) *VEGFA* and (**d**) *FGFR1*, respectively. *x*-axis data points at probe level plotted from chromosome 1 to 22 and *x* with boundaries of chromosomes indicated by vertical-dotted lines. *y*-axis, log2 ratios.

Amplifications in cancer are of special interest as these may contain driver oncogenes. Across the present series of 349 primary CRC tissue samples, in total 432 amplifications were detected. An overview of the top 25 most frequent amplifications identified, along with the genes located at these loci, is presented in Table 1. Forty-three out of these 80 genes have previously been associated with cancer in general and/or with CRC in particular^11^,^12^,^15^. In addition amplifications of the genes *VEGFA* and *EGFR* were found in, respectively, 2 and 1 samples from the 349 patients. One of the most appealing aspects of amplifications is their possible association to drug therapy, with trastuzumab sensitivity in *ERBB2*(Her2neu) amplified breast cancer as a classic example. Therefore, gene targets of both Food and Drug Administration (FDA)-approved drugs as well as drugs under (pre-) clinical evaluation were mapped to the genes in the amplifications identified (Table 2). Moreover, the list of amplified genes also contained kinases, ligands and receptors that have not yet been targeted (*n*=25; Supplementary Tables 6–8). Examples of DNA copy number profiles with amplified regions containing genes including drug targets are shown in Fig. 7. Among the signal transduction pathways, the *RAS, RAF* and *ERK* pathway^23^ is of particular interest in mCRC, given the role of anti-*EGFR* therapy in these patients. Seventeen genes in this pathway, ranging from ligands to downstream targets, were found to be amplified, of which *AKT5* and *MYC* occurred in five of the patients. Amplification of *KRAS* occurred in two patients and *ERBB2* in three patients (Supplementary Table 9).

## Discussion

The diversity of the genomic landscape in CRC overall has been^12^,^13^,^15^ and still is under active investigation, for example, in the context of TCGA and the International Cancer Genome Consortium (ICGC)^24^. These efforts have painted a canvas of the genomic diversity in CRC, ranging from the ‘Vogelgram’ model of somatic mutations in the pathogenesis of CRC to the ‘mountains and hills’ concept^2^,^12^. Next to that, also evident patterns of DNA copy number aberrations as well as DNA methylation changes have been documented^25^,^26^. The challenge is to associate this genomic diversity to phenotypic characteristics, such as response to systemic treatment. The field of somatic genomic variation, with neoplasia as main associated phenotype, is substantially lagging behind that of germ-line genomic variation, that is, clinical genetics and molecular epidemiology. An important lesson from this latter field teaches us that homogeneity of phenotype is a key factor in genome-wide association studies. This is eminent from the fact that many important genomic associations actually have been discovered in inbred populations. This puts high demands on the quality and level of detail of phenotypic annotation, to such an extent that so far in a multicentre setting this almost only has been feasible in prospective studies like phase III clinical trials, examples of which are PETACC-3 (ref. 16) and the present study. Similar demands go for the samples studied and the genomic read-out systems used. As to the latter, the aim is to capture as much as possible of the spectrum genomic aberrations in the tumour samples, under the restrictions imposed by sample types available. For many tumour types this limits methods to those that can be applied to the FFPE archival material harvested from the resection specimen. In practice, this restricts the read out to the DNA level, and aCGH still is a widely validated technology for capturing genome-wide variation from FFPE samples^20^,^27^. The existence of an operational national pathology archive further facilitated the retrieval and processing of the resection samples of the current study under thorough quality control^28^. Patients studied had participated in either of the two Dutch multicentre mCRC phase III clinical trials, that is, CAIRO (ref. 17) and CAIRO2 (ref. 18), and FFPE tissues were analyzed for associations between somatic genomic variation and clinical phenotype, that is, between high-resolution genome-wide DNA copy number aberrations and PFS to first-line palliative systemic therapy. This study yielded many promising associations between DNA copy number aberrations and response to first-line drug therapy within and between treatment arms. Three chromosomal regions showed statistically promising associations for patients treated with or without irinotecan. Irinotecan sensitivity could be validated through association with DNA copy number aberrations of cell lines from the COSMIC^21^. Consistent with the observations we made with the clinical samples, a significant association with viability for all cell lines, as well as for the CRC cell lines alone was observed for one of the three chromosomal regions of interest, namely gain of 6q16.1−q21. Other findings reported here still await external validation, and at this point in time those results should be considered hypothesis generating. Yet, the release of this large body of high-quality data, both in terms of phenotype annotations and genomic read out, to the wider scientific community will provide the best chance of such validation.

In addition to studying the associations of genome-wide DNA copy number aberrations to PFS for three different drug regimens in mCRC, we also documented the prevalence of amplifications in mCRC. These amplifications, that apparently are associated with a selection advantage for the prevailing tumour clone in the individual patient, may give rise to overexpression of oncoproteins that are targets of currently available drugs. The classical example of this is amplification of Her2neu (*ERBB2*) in breast cancer, but that also was present in three of the CRC samples studied. Genes in amplified regions included several known targets for FDA-approved drugs and potential targets of therapeutic interest were identified. We hypothesize that amplification of drug targets in these tumours may be of help for treatment selection in individual patients. *FGFR1*, a known target receptor of pazopanib and regorafenib, showed high-level gains in 3.7% of patients in the present study and might represent an appealing example, since a recent report indicated that treatment with regorafenib results in a modest PFS and overall survival benefit in unselected patients with refractory mCRC compared with placebo^29^.

In the present study, chromosomal copy number aberrations from primary tumours rather than from the actual metastases were analyzed, even though the goal was to associate disease biology to response to drug therapy for metastatic disease. One may argue that the clones that have metastasized would differ substantially from the primary tumour. While such changes do occur, and secondary mutations may contribute to drug resistance, like in gastrointestinal stromal tumour and non-small cell lung cancer, primary tumours and their metastases share the vast majority of genetic aberrations, which of course is not surprising for an evolutionary process. We recently demonstrated that DNA copy number aberrations mostly persist in CRC metastases^30^, which is consistent with *KRAS* mutation status in primary and mCRC^31^. Differences between tumours from different patients are greater than between primary tumours and their metastases^30^, which is consistent with the predestination hypothesis that poses the full genomic programme that determines if the biological and clinical phenotype of a tumour is already present at the time when a primary cancer arises^32^. Whether patients with liver metastases will benefit from a given drug therapy will largely depend on the biological characteristics of their disease. Given the high level of conservation of genomic characteristics of the primary tumour in the associated metastasis, answer to this clinically relevant question indeed may come from analysis of the primary tumour tissue, as also has been well-demonstrated for *KRAS* mutation analysis in case of anti-EGFR therapy^3^.

Inherent to the search for biological subgroups of any type of cancer that may differ in their response to drug therapy is the fact that such subgroups may represent rather small proportions of the overall disease phenotype. This poses a challenge to the statistical strategies we use to exclude the risk of scientific observations being merely due to chance. Statistical methods available to estimate the probability of these so called type I errors have their optimal performance when variant subsets are of equal and sufficient size, and consequently may overestimate the probability of associations involving small subgroups being due to chance. Evaluation of descriptive data like Kaplan–Meier curves, however, showed several instances of infrequent aberrations with relatively strong associations. This is an indication that genomic diversity in CRC is greater than anticipated, and that these small genomic hills still may be highly relevant in a clinical setting. Therefore an urgent need exists to design and organize studies for validating such observations in a way in which they can provide solid ground for clinical decision making.

## Methods

### Sample selection

Patients selected for the current study participated in either of the two multicentre phase III trials of the Dutch Colorectal Cancer Group (DCCG), namely CAIRO (CKTO 2002-07, ClinicalTrials.gov; NCT00312000) and CAIRO2 (CKTO 2005-02, ClinicalTrials.gov; NCT00208546). The two randomized clinical trials were approved by the Committee on Human-Related Research Arnhem—Nijmegen and by the local institutional review boards. The written informed consent required for all patients before study entry also included translational research on tumour tissue. In the CAIRO study, 820 patients with mCRC without prior palliative systemic treatment were randomized between sequential (arm-A, first-line CAP, second-line irinotecan and third-line CAPOX) and combination treatment (arm-B, first-line CAPIRI and second-line CAPOX)^17^. In the CAIRO2 study, 755 patients were randomly assigned to first-line treatment with capecitabine, oxaliplatin and bevacizumab (arm-A, CAPOX-B), or the same regimen combined with cetuximab (arm-B)^18^. From CAIRO2, only samples from patients in arm-A were included, since a worse PFS for patients in arm-B was observed, which may have been caused by a negative interaction between bevacizumab and cetuximab. In the present study we included patients in whom FFPE tissue of the primary tumour as well as matched normal DNA for reference purposes was available through the national pathology registry PALGA^28^. Inherently these are the patients who underwent resection of the primary tumour, of whom 311, 322 and 282 patients were included from the CAP, CAPIRI and CAPOX-B treatment groups, respectively. Of these, tissue samples were available for 221 patients receiving CAP, 257 patients receiving CAPIRI and 191 patients receiving CAPOX-B.

Stringent criteria were used to select patients based on tumour cell percentage of the tissue sample, clinical variables, DNA quality and quality of the resulting copy number profiles (Fig. 1). Haematoxylin and eosin-stained tissue sections were reviewed by a pathologist to determine the percentage of tumour cells. Only tumours containing an area of at least 70% tumour cells were selected for DNA extraction. After exclusion of samples with insufficient tumour cell content, 188, 178 and 158 samples from the CAP, CAPIRI and CAPOX-B patient groups, respectively, were available for further analysis. From these, 109 samples were selected from the CAP, 115 from the CAPIRI and 141 from the CAPOX-B treatment group. The patient samples were matched according to the stratification factors in the original studies (for the subgroup of patients that underwent resection of the primary, since these are the patients from whom material was available to be included in this study), that is, performance status, predominant metastatic site, previous adjuvant therapy and serum lactate dehydrogenase level (LDH) in CAIRO (CAP and CAPIRI) and number of affected organs, previous adjuvant therapy and serum LDH in CAIRO2 (CAPOX-B).

### DNA isolation, labelling and quality assessment

For the selected samples, tumour and matched normal reference DNA was isolated from FFPE tissue blocks as described previously^20^. Haematoxylin and eosin sections of 3–5 μm thickness were cut prior to the sections used for DNA isolation^33^. Four to six 10 μm adjacent sections were cut, de-paraffinized, and macro dissected. DNA was extracted using a column-based method (QIAamp microkit; Qiagen, Hilden, Germany)^34^. Labelling was performed using the Enzo Genomic DNA Labeling kit according to the manufacturer’s instructions (Enzo Life Science, Farmingdale, NY, USA)^35^ and purified using the QIAGEN MinElute PCR Purification Kit with an elution volume of 2 × 10.5 μl. (Qiagen, Westburg, Leusden, Netherlands). Quality of labelled DNA was tested with the NanoDrop 1,000 (Thermo Fisher Scientific, Delaware, USA) by measuring the specific activity (pico mole dye per microgram genomic DNA). If for either tumour or normal reference a specific activity <16 pmol μg^−1^ was measured, samples were considered insufficient and were replaced by clinically comparable samples. If both tumour and reference were between 12 and 16 pmol μg^−1^, but had equal specific activity, they were not excluded. This method of DNA quality testing was extensively examined by hybridizing samples with decreasing specific activity. The resulting DNA copy number profiles were evaluated by visual inspection and the MAD value^34^. Tumour and matched normal reference samples with a specific activity of at least 16 pmol μg^−1^ turned out to result in good quality copy number profiles. In addition tumour and matched normal reference samples with equal specific activity >12, but <16 pmol μg^−1^, turned out to result in copy number profiles of sufficient quality. Fourteen, 21 and 6 samples in the CAP, CAPIRI and CAPOX-B patient groups, respectively, did not pass the DNA quality test and were replaced by other samples of which in the end 11, 19 and 0 samples passed the test.

### Array hybridizations

In total, 112, 113 and 136 tumour and matched normal reference samples were hybridized on the 4 × 180K customized Agilent oligonucleotide arrays (Agilent Technologies, Palo Alto, CA, USA). These arrays contain 180,880 *in situ* synthesized 60-mer oligonucleotides, representing 169,793 unique chromosomal locations distributed across the genome at ~17 kb intervals and is enriched with 4,548 additional unique oligonucleotides, located at 238 of the Cancer Census genes^11^. The exact array design can be found in the Gene Expression Omnibus (GEO)^36^ platform GPL8687 (http://www.ncbi. nlm.nih.gov/geo). Labelling was performed using the Enzo Genomic DNA Labeling kit according to the manufacturer’s instructions (Enzo Life Sciences, Raamsdonksveer, Netherlands). Cy3- and Cy5-labelled DNA samples were combined with Cot-1 DNA (Invitrogen, Breda, Netherlands) and blocking agent in hybridization buffer (Agilent Technologies). Hybridization mixture was heated for 3 min at 95 °C and subsequently incubated for 30 min at 37 °C. Hybridization was performed for 24 h at 65 °C followed by 5 min washing with wash buffer 1 at room temperature (RT), and 1 min with wash buffer 2 at 37 °C and 1 min with acetonitrile at RT^35^. Microarray scanner G2505C (Agilent technologies) was used for scanning, and feature extraction software (version 10.5.1.1; Agilent Technologies, protocol CGH_105_Dec08) was applied using default settings. To keep all experiments comparable, no quality flagging was applied and all oligonucleotides were included in the downstream analysis. The oligonucleotides were mapped along the genome according to the NCBI36/hg18 built (March 2006). Of both Cy3 and Cy5 channels, local background was subtracted from the median intensities. The log2 ratios were calculated and normalized by subtraction of the median value of all probes spotted on the array. Normality of the matched normal reference DNA was evaluated *in silico* by comparison with a reference pool of normal FFPE DNA, using the across array approach^35^. DNA copy number profiles with MAD values >0.4 were excluded. For technical reasons, for five samples, the matched normal reference was replaced by a reference pool of normal FFPE DNA *in silico*, using the same across array approach^35^.

### DNA copy number data pre-processing

Genome data analysis was performed in the programming language R 2.9.1. The aCGH profiles were first dewaved^37^ and then the median was normalized. For segmentation, the Bioconductor R-package DNAcopy version 1.22.1 (ref. 38) was applied followed by post segmentation mode normalization. Profiles were corrected for tumour cell percentage, and DNA copy number calls for loss, normal, gain or amplification were made using the using R-package CGHcall version 2.8.0 (ref. 39), applying posterior probabilities of 0.5 or higher. Amplifications were furthermore defined by a chromosomal size of 3 Mb or less according to Leary *et al*.^13^ Accuracy of normalization, segmentation and calling was verified by visual inspection. To reduce dimensions of the aCGH data set without losing information, the information of the 180.000 probes was reduced to 855 chromosomal subregions using the Bioconductor R-package CGHregions version 1.6.0 (ref. 40) and contains all the genomic variation in this data set of 349. For functional validation purposes, chromosomal subregions that significantly associated with PFS (uncorrected single-test *P*-values<0.005) were concatenated to a ‘total’ chromosomal region if they occurred within one chromosomal arm.

### Downstream analysis and integration with clinical data

Regions of gains and losses were used for supervised and unsupervised analysis. The R-package WECCA version 0.30 (ref. 41), designed for clustering copy number profiles rather than expression profiles, was used with probabilities as described by Smeets *et al*.^42^ with settings: ‘ordinal’, ‘all equal’ and ‘Ward linkage’. For supervised analysis, only those patients were included who received at least three treatment cycles or two cycles if patients died due to (rapidly) progressive disease. In addition, microsatellite instable samples, as determined previously^43^, were excluded since they showed infrequent copy number aberrations. In total, 92, 94 and 119 patients treated with CAP, CAPIRI and CAPOX-B, respectively were included. A log-rank test using 10.000 permutations was performed to calculate the significance of DNA copy number correlations to PFS of the first-line treatment in each study arm^44^ (uncorrected single-test *P*-values<0.005, corresponding to permutation-based False Discovery Rates in the range 20–30%). Separate analyses were run on gains or losses to test for associations with PFS.

### Gene identification

UCSC genes (hg18; ref. 45) were mapped to the genomic regions that showed an association to PFS with a *P*-value<0.05. For gene identification, the association of DNA copy number aberrations with mRNA expression was evaluated using publicly available data from TCGA 8 July 2011 update^15^,^46^. This set consisted of 141 CRCs for which both DNA copy number data (obtained with the Affymetrix SNP 6.0 platform) and mRNA expression data (Agilent G4502A platform) were available. To further narrow down at the gene level, only those genes were considered that were located on regions that showed an association to PFS with a *P*-value<0.005 in any arm, or with a *P*-value<0.05 in at least two arms and that showed a positive correlation between DNA copy number status and mRNA expression. To investigate genes with a possible role in drug pathways, the Pharmacogenomics Knowledge Base^23^,^47^,^48^,^49^,^50^ (http://www.pharmgkb.org/) was used. Furthermore, genes identified in our study were overlaid with genes identified in other landscape papers, including ‘The genomic landscapes of human breast and CRCs’ and the TCGA data set^12^,^15^.

Drug target genes were extracted from this list with Ingenuity Pathway Analysis version 8.7 (IPA, http://www.ingenuity.com/) taking all drug target genes, regardless of drug class, into account. Drug target genes that were located at amplified chromosomal loci were ordered according to the indication and mechanism of action of the drug associated with this specific gene. If multiple drugs were listed by IPA for targeting a specific gene, a maximum of three drugs were shown in the corresponding tables of this paper, giving priority to FDA-approved drugs that are most frequently used and representative of the drug class as well as agents furthest in (pre-) clinical development. Potential drug target genes were also selected by searching for known receptors and ligands in the Database of Interacting Proteins (http://dip.doe-mbi.ucla.edu/dip/). In addition, we have identified genes coding for kinases by categorizing the original gene list in IPA version 8.7.

### Drug sensitivity validation data and procedures

Chromosomal copy number data from 1,005 cell lines were downloaded from the COSMIC^21^ on 25 April 2014 via the URL http://cancer.sanger.ac.uk/cancergenome/projects/cell_lines. These data had been generated using Affymetrix SNP 6.0 arrays (1.8 million genetic markers, GEO accession number: GPL6801; Affymetrix, Santa Clara, USA) and processed using PICNIC^51^. Chromosomal regions of copy number >2, gain, =2, normal (diploid) and <2, loss, were inferred (mapped to NCBI37/hg19). The half maximal inhibitory concentration (IC50) values for SN-38 (7-Ethyl-10-Hydroxy-Camptothecin), the active metabolite of irinotecan were available for 578 cell of the COSMIC lines and were downloaded via the Sanger Welcome Trust-GDSC project portal http://www.cancerrxgene.org^22^ on 25 April 2014. No IC50 data were available for 5 fluorouracil/capecitabine, oxaliplatin or bevacizumab. Chromosomal subregions for significant associations with PFS were observed in the patient samples (Supplementary Table 2), were concatenated if they were adjacent and occurred within one chromosomal arm, thereby reducing the number of regions to seven main chromosomal regions, three of which were significantly correlated with irinotecan treatment. Coordinates of these three regions were mapped to NCBI37/hg19 using the liftOver tool at UCSC (http://hgdownload.cse.ucsc.edu/admin/exe). Cell lines were classified for each of the three regions as aberrant (gain or loss) for any overlap. A Kruskal–Wallis test was applied per region to assess if the distribution of IC50 values for cell lines with a chromosomal aberration (gain or loss) was significantly different from those without a chromosomal aberration (normal), for all cell lines (*n*=573) and the CRC cell lines alone (*n*=31). Only three regions were tested, hence uncorrected *P*-values≥0.05 were considered statistically significant.

### Data analysis strategy

Discovery of DNA copy number aberrations associated with PFS under the three drug regimens was performed on the aCGH data from the CAIRO (CAP and CAPIRI) and CAIRO2 (CAPOX-B) samples. For biological validation, the association of DNA copy number aberrations of the genes involved with mRNA expression was evaluated using publicly available data from TCGA^15^,^46^. This data set consisted of 141 CRCs for which both DNA copy number data (obtained with the Affymetrix SNP 6.0 platform) and mRNA expression data (obtained with the Agilent G4502A platform) were available.

## Author contributions

D.I., H.F.v.E., Q.J.M.V. and X.Q. carried out the experiments. J.C.H., M.L., M.K., J.T., L.J.M.M., M.A.vd.W., N.C.T.v.G., L.J.W.B., C.R., O.K., H.M.W.V., I.D.N., C.J.A.P., B.Y. and G.A.M. analyzed and interpreted the data. J.C.H., M.A.vd.W., C.J.A.P., B.Y. and G.A.M. were responsible for the study design. J.C.H., M.L., B.Y. and G.A.M. wrote the paper. All authors revised the manuscript critically and were involved in editing the paper and final approval of the submitted and published versions. The funders had no role in the study design, data collection, data analysis, decision to publish or preparation of the manuscript.

## Additional information

**How to cite this article:** Haan, J. C. *et al*. Genomic landscape of metastatic colorectal cancer. *Nat. Commun.* 5:5457 doi: 10.1038/ncomms6457 (2014).

**Accession codes:** Array data have been deposited in the gene expression omnibus (GEO) under the accession code GSE36864.

## Supplementary Material

Supplementary Information Supplementary Figures 1-11, Supplementary Tables 1-9 and Supplementary References

## Figures and Tables

**Table 1:** Overview of top 25 most frequent amplifications identified by high-resolution aCGH in 349 primary tumour samples of colorectal cancer patients that ultimately developed distant metastases.

**Chr**	**Band**	**Start (kb)**	**End (kb)**	**Frequency**	**Genes at the locus of amplification**
8	8p23.1	7729.31	7747.83	6	*SPAG11A*
8	8p12	38056.98	38195.55	4	*ASH2L STAR LSM1 BAG4*
8	8p12	38295.33	38410.39	7	LETM2 FGFR1*
8	8p11.23	39356.6	39402.49	5	
8	8p11.23	39464.58	39482.04	6	
8	8p11.21	41468	41567.59	5	*GOLGA7 GINS4 AGPAT6*
8	8q24.21	128387.5	129035.7	5	DQ515898 DQ515899 DQ515897 POU5F1* MYC* *PVT1 TMEM75*
11	11q14.3	89402.45	89457.56	4	*TRIM49*
12	12p13.31	9554.15	9582.76	15	
13	13q12.13	26419.12	26496.5	4	
13	13q12.2	27366.6	27711.8	5	PDX1 CDX2* PRHOXNB FLT3* *PAN3*
13	13q21.31	63231	63293.05	4	
13	13q22.1	72712.75	72880.71	6	
18	18q21.1	44805.82	44837.45	4	
18	18q21.1	45094.24	45159.05	4	
19	19q13.2	44256.77	45231.21	5	*PAK4 NCCRP1 SYCN IL28B IL28A IL29 LRFN1 GMFG SAMD4B PAF1 MED29 CR601007 ZFP36 PLEKHG2 RPS16 SUPT5H TIMM50 DLL3 EID2B EID2 LGALS13 AK023628 LGALS14 CLC DYRK1B FBL FCGBP PSMC4 ZNF546*
19	19q13.2	45355.25	45544.88	5	MAP3K10 TTC9B CNTD2 AKT2* *C19orf47*
20	20q13.11–q13.12	41179.95	41732.85	4	*SFRS6*† *L3MBTL SGK2 IFT52 MYBL2*
20	20q13.12	42494.6	42562.9	4	*C20orf62 TTPAL SERINC3*
20	20q13.32	56021.92	56663.86	5	*C20orf85 PPP4R1L RAB22A VAPB APCDD1L AK091704 AK054637 STX16*
20	20q13.33	61334.28	61398.13	5	*BIRC7 NKAIN4 ARFGAP1 COL20A1*
20	20q13.33	62202.02	62351.03	6	*OPRL1 NPBWR2*† *MYT1*
23	Xq21.31	88529.93	88545.42	4	
23	Xq21.31	88563.15	88563.21	4	
23	Xq21.31	89760.38	89824.39	4	

aCGH, array comparative genomic hybridization; Chr, chromosome.

^*^Cancer census genes^11^.

^†^Mutated in data from the ‘The genomic landscapes of human breast and colorectal cancers’^12^.

**Table 2:** Drug target genes at amplifications in 349 primary tumour samples of patients that ultimately developed distant metastases.

**GeneID**	**Chr**	**Frequency**	**Drug**	**Drug class**
*Clinically available targeted anticancer agent-related genes*				
*FGFR1*	8	8	Pazopanib	TKI
*FLT3*	13	5	Sorafenib, sunitinib	TKI
*FLT1*	13	4	Sunitinib, pazopanib, axitinib	TKI
*HSP90AB1*	6	2	17-DMAG, IPI-504 (retaspimycin)	HSP90 inhibitor
*VEGFA*	6	2	**Bevacizumab**, ranibizumab, aflibercept, pegaptanib	Anti-vegf MAb; vegf receptor decoy; anti-vegf aptamer
*CDK8*	13	1	Flavopiridol	Anti-cdk flavonoid
*Cytotoxic drug-related genes*				
* ADA*	20	3	Pentostatin, vidarabine	Antimetabolite
* TUBB1*	20	2	Docetaxel, epothilone B; vinorelbine	Taxane; vinca-alkaloid
*Target genes of drugs for other indications*				
* DGAT1*	8	3	Omacor	Antilipemic
* HRH3*	20	3	Tesmilifene, triprolidine, buclizine	Antihistamine/anticholinergic
* FNTA*	8	2	Lonafarnib, tipifarnib	Farnsesyl transferase inhibitor
* CD40*	20	1	SGN-40 (dacetuzumab)	Anti-hucd40 MAb
* TNFSF13B*	13	1	Belimumab	BLyS-specific inhibitor
*Target genes for drugs without apparent anticancer activity*				
* OPRL1*	20	6	ZP120	
* CHRNA4*	20	4	Pancuronium	
* OGFR*	20	3	Enkephalin, methionine	
* NTSR1*	20	3	Contulakin-G	
* COL9A3*	20	3	Collagenase clostridium histolyticum	
* CHRNB3*	8	2	Pancuronium	
* CHRNA6*	8	2	Pancuronium	
* F10*	13	1	Dalteparin, heparin, enoxaparin	
* COL4A2*	13	1	Collagenase clostridium histolyticum	
* F7*	13	1	Nematode anticoagulant protein c2	
* PDE7A*	8	1	Anagrelide, tolbutamide, theophylline	
* AHCY*	20	1	3-deazaneplanocin, neplanocin A	
* COL4A1*	13	1	Collagenase clostridium histolyticum	

Chr, chromosome; MAb, monoclonal antibody; TKI, tyrosine-kinase inhibitor.

Genes are ordered as targets of targeted anticancer agents, cytotoxic drugs, drugs approved for indications other than cancer and drugs without known anticancer effects.
