# Supplementary material for: Genomic landscape of metastatic colorectal cancer
Source: Nat Commun. 2014 Nov 14;5:5457. doi: 10.1038/ncomms6457 (PMC4243240; doi:10.1038/ncomms6457)
Supplement: Supplementary Information — Supplementary Figures 1-11, Supplementary Tables 1-9 and Supplementary References [file ncomms6457-s1.pdf]

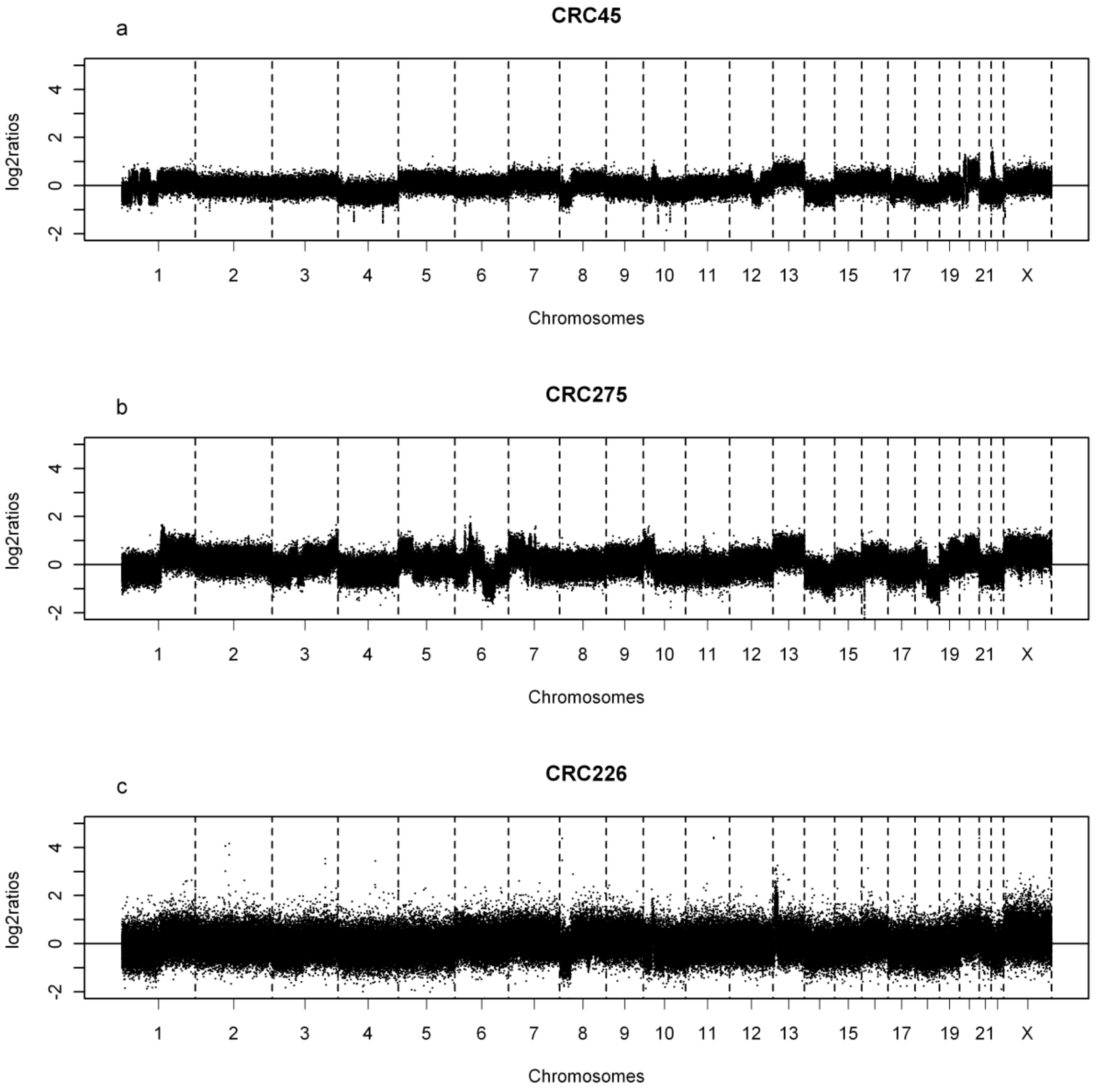

Supplementary Figure 1: Representative examples of DNA copy number profiles with variable MAD values a) MAD=0.16, b) MAD= 0.24 and c) MAD=0.39. On the horizontal axis the data points at probe level are plotted from chromosome 1 to chromosome 22 and X. On the vertical axis log2 ratio's are depicted. Boundaries of chromosomes are indicated by dotted lines.

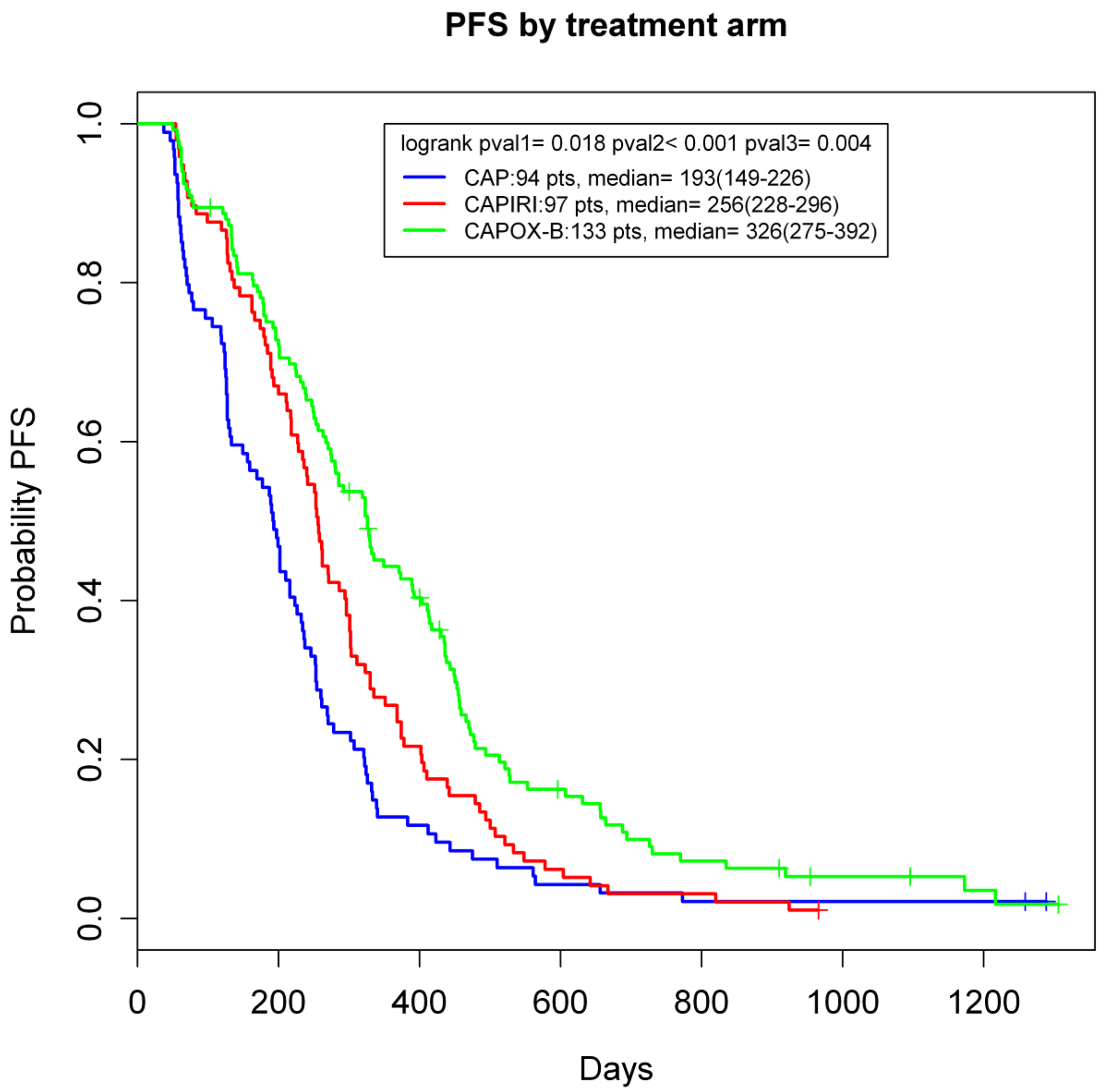

Supplementary Figure 2: Kaplan–Meier PFS analysis of patients with advanced colorectal cancer in the three treatment groups

The group receiving CAP depicted in blue, CAPIRI in red and CAPOX-B in green. Logrank pvalues, p<0.005 are considered significant; pval1, CAP versus CAPIRI; pval2, CAP versus CAPOX-B; pval3, CAPIRI versus CAPOX-B; pts, number of patients; median, median survival with confidence limits within brackets.

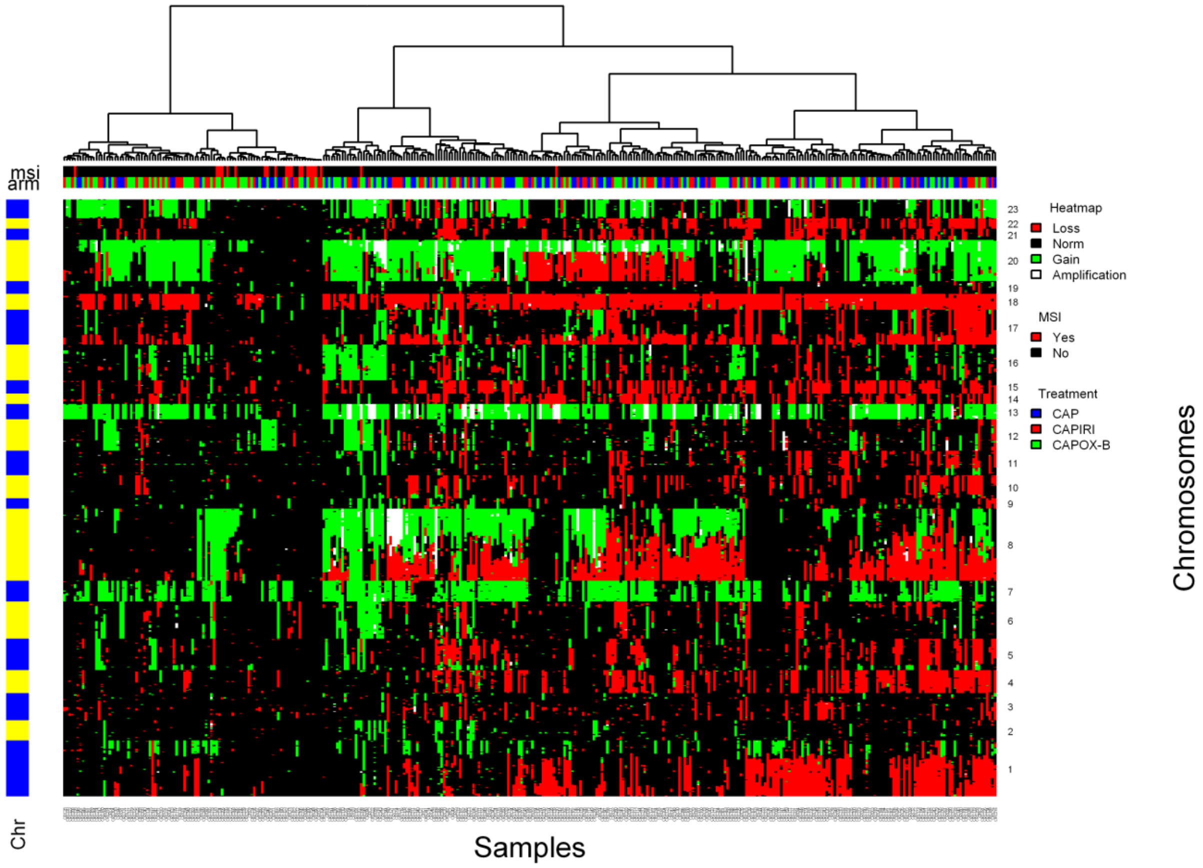

Supplementary Figure 3: Dendrogram of unsupervised hierarchical clustering of all 349 tumors

Samples on the horizontal axis and chromosomal regions on the vertical axis, ordered by chromosomal position. MSI tumors in red, MSS tumors in black, patients treated with CAP in blue, CAPIRI in red, CAPOX-B in green. Odd chromosomes are depicted in blue and even chromosomes in yellow. Within the heatmap, black blocks depict normal copy number, red blocks loss and green blocks gain and white blocks amplification.

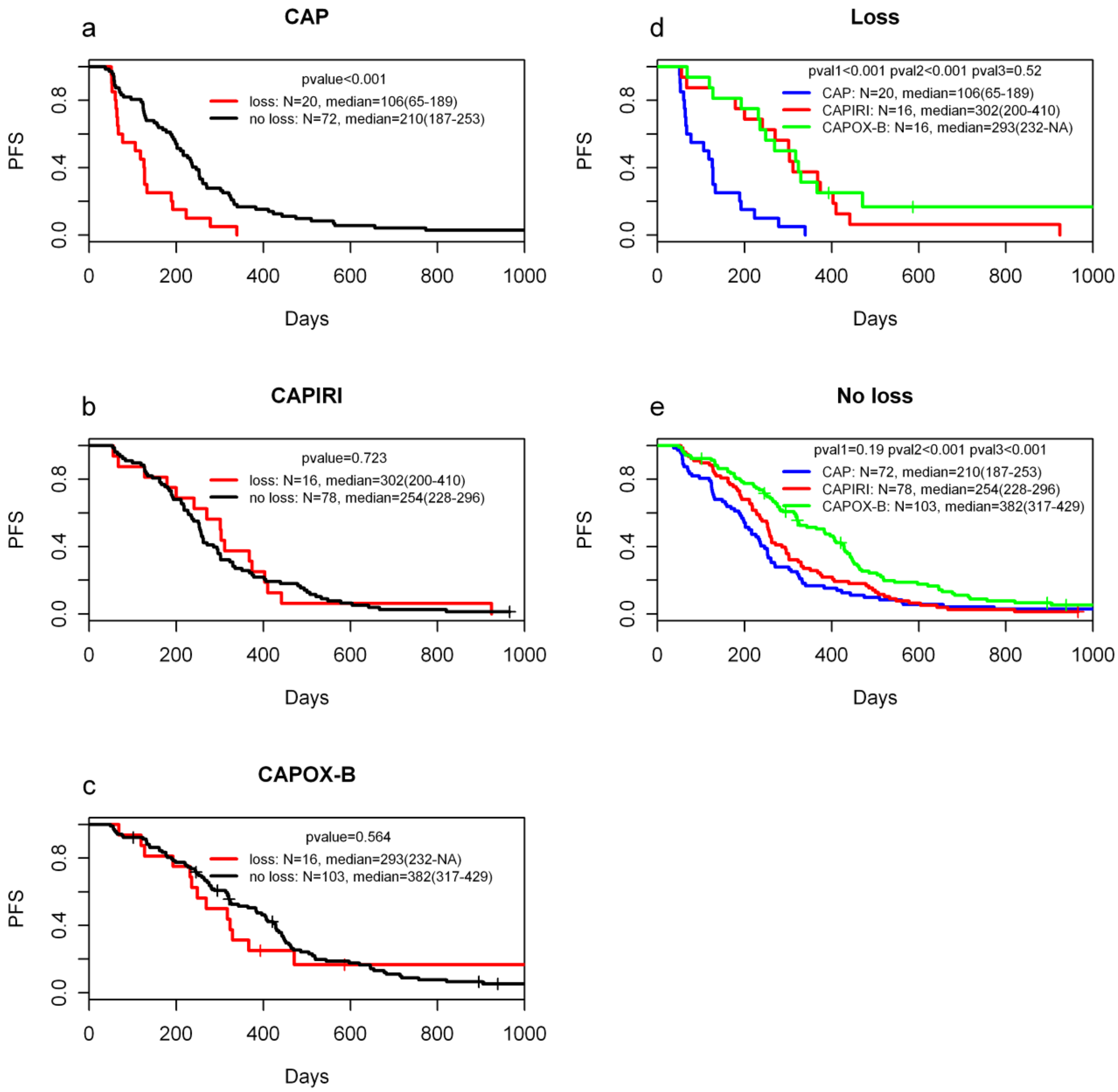

Supplementary Figure 4: Kaplan–Meier PFS analysis for chromosomal sub-region 5q12.1-q12.3

Within patients groups a) CAP; b) CAPIRI; c) CAPOX-B, with loss in red, without loss in black. Between patient groups d) loss of 5q12.1-q12.3; e) no loss of 5q12.1-q12.3, with CAP in blue, CAPIRI in red, CAPOX-B green. Log-rank p-values, p<0.005 are considered significant; pval1, CAP versus CAPIRI; pval2, CAP versus CAPOX-B; pval3, CAPIRI versus CAPOX-B; N, number of patients; median, median survival with confidence limits within brackets and NA, no applicable value.

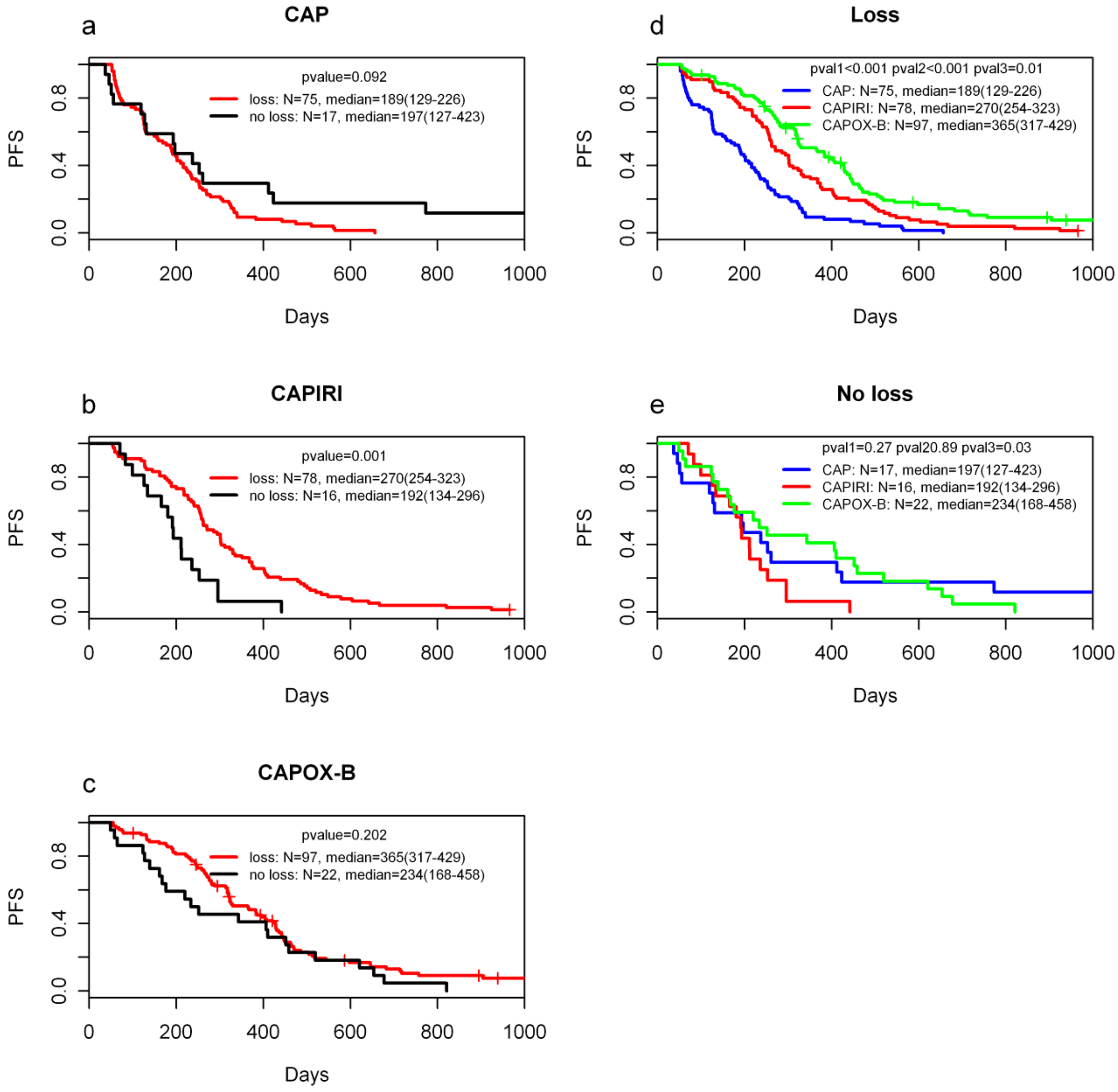

Supplementary Figure 5: Kaplan–Meier PFS analysis for chromosomal sub-region 18q21.33-q22.3
Within patients groups a) CAP; b) CAPIRI; c) CAPOX-B, with loss in red, without loss in black. Between patient groups d) loss of 18q21.33-q22.3; e) no loss of 18q21.33-q22.3, with CAP in blue, CAPIRI in red, CAPOX-B green. Logrank pvalue, Log-rank p-values, p<0.005 are considered significant; pval1, CAP versus CAPIRI; pval2, CAP versus CAPOX-B; pval3, CAPIRI versus CAPOX-B; N, number of patients; median, median survival with confidence limits within brackets and NA, no applicable value.

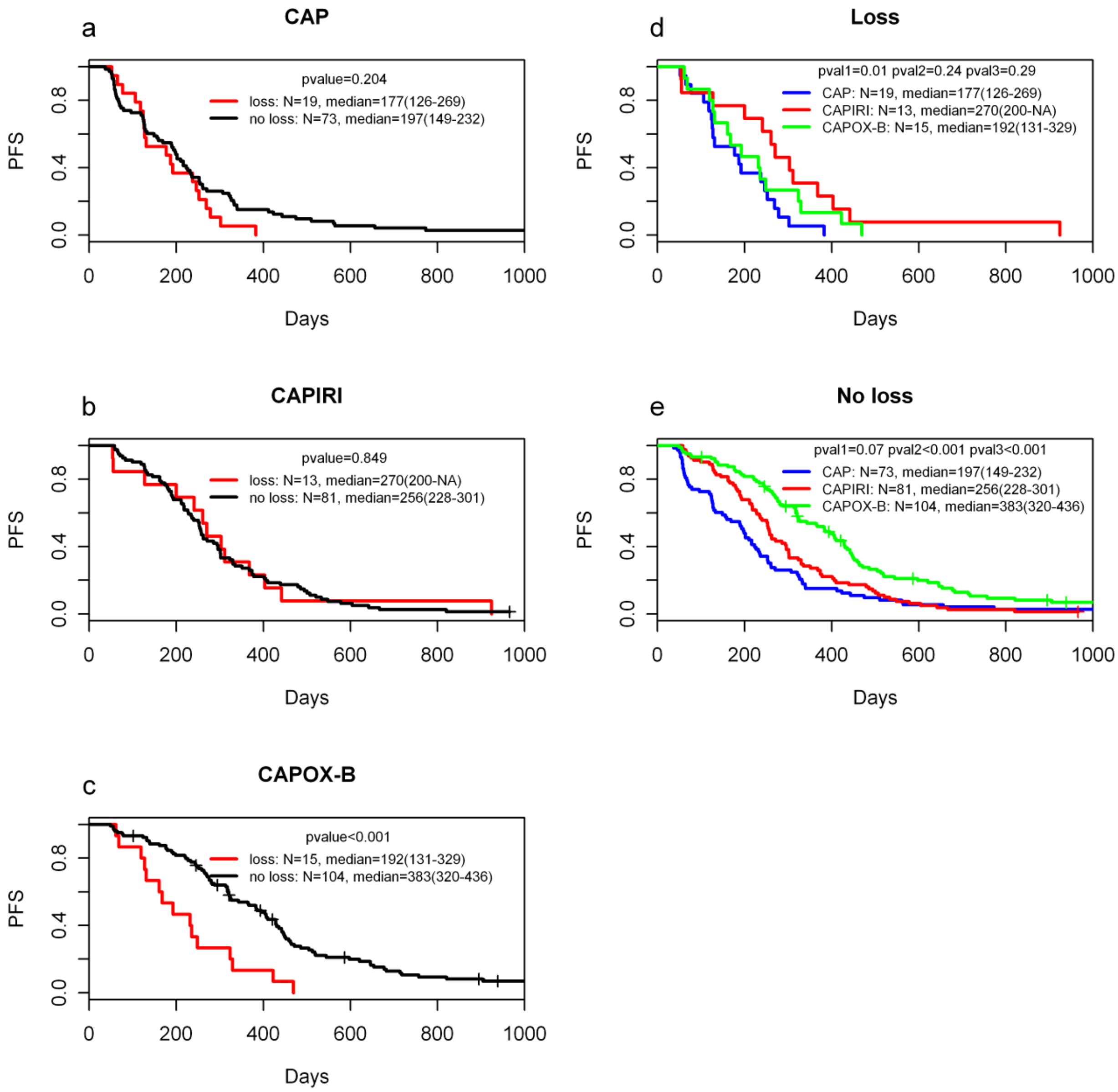

Supplementary Figure 6: Kaplan–Meier PFS analysis for chromosomal sub-region 5q34

Within patients groups a) CAP; b) CAPIRI; c) CAPOX-B, with loss in red, without loss in black. Between patient groups d) loss of 5q34; e) no loss of 5q34, with CAP in blue, CAPIRI in red, CAPOX-B green. Log-rank p-values, p<0.005 are considered significant; pval1, CAP versus CAPIRI; pval2, CAP versus CAPOX-B; pval3, CAPIRI versus CAPOX-B; N, number of patients; median, median survival with confidence limits within brackets and NA, no applicable value.

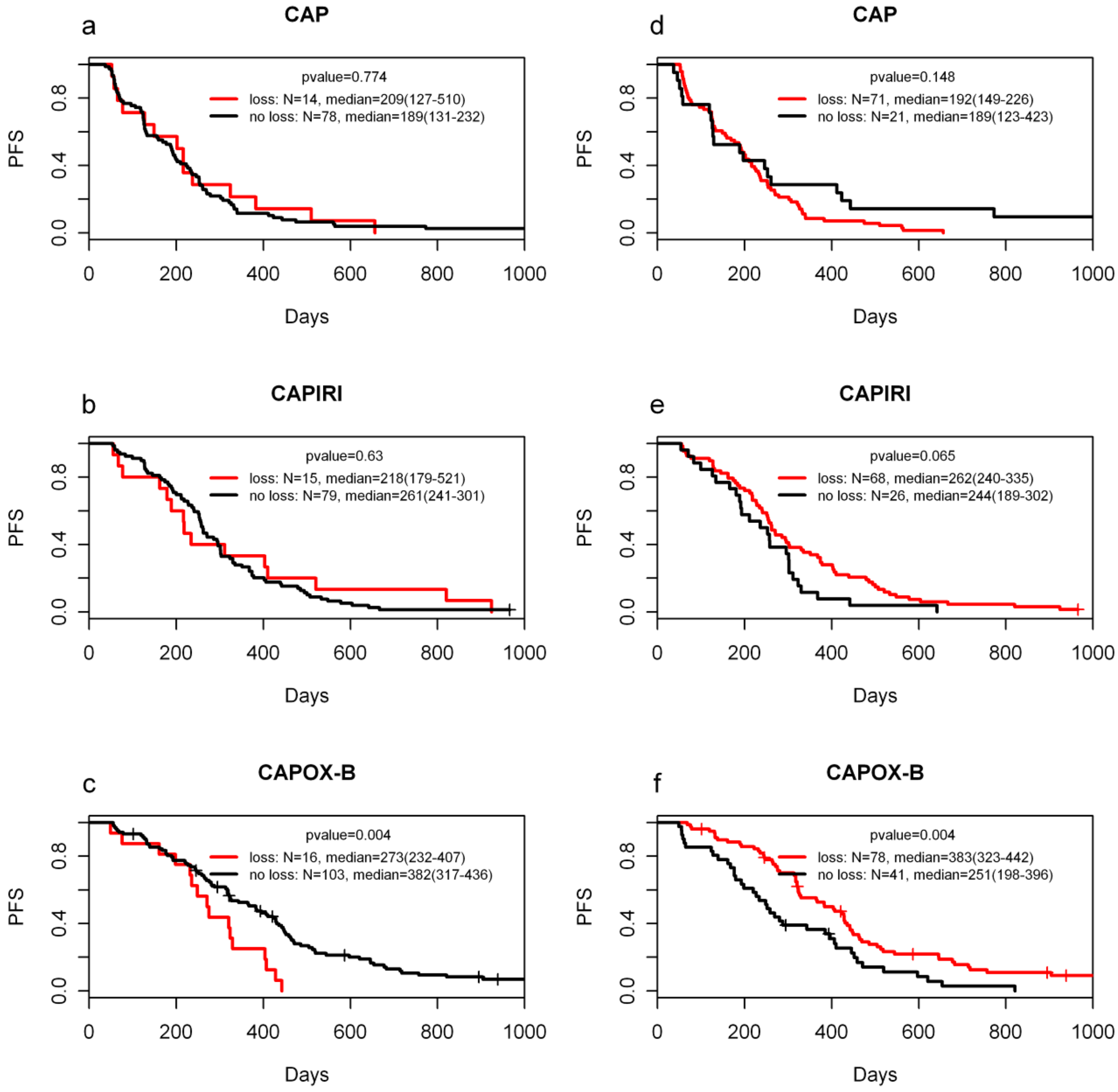

Supplementary Figure 7: Kaplan–Meier PFS analysis for chromosomal sub-regions 17q12-q21.31 and 18q11.2 Within patients groups a and d respectively) CAP; b and e respectively) CAPIRI; c and f respectively) CAPOX-B, with loss in red, without loss in black. Log-rank p-values, p<0.005 are considered significant; N, number of patients; median, median survival with confidence limits within brackets and NA, no applicable value.

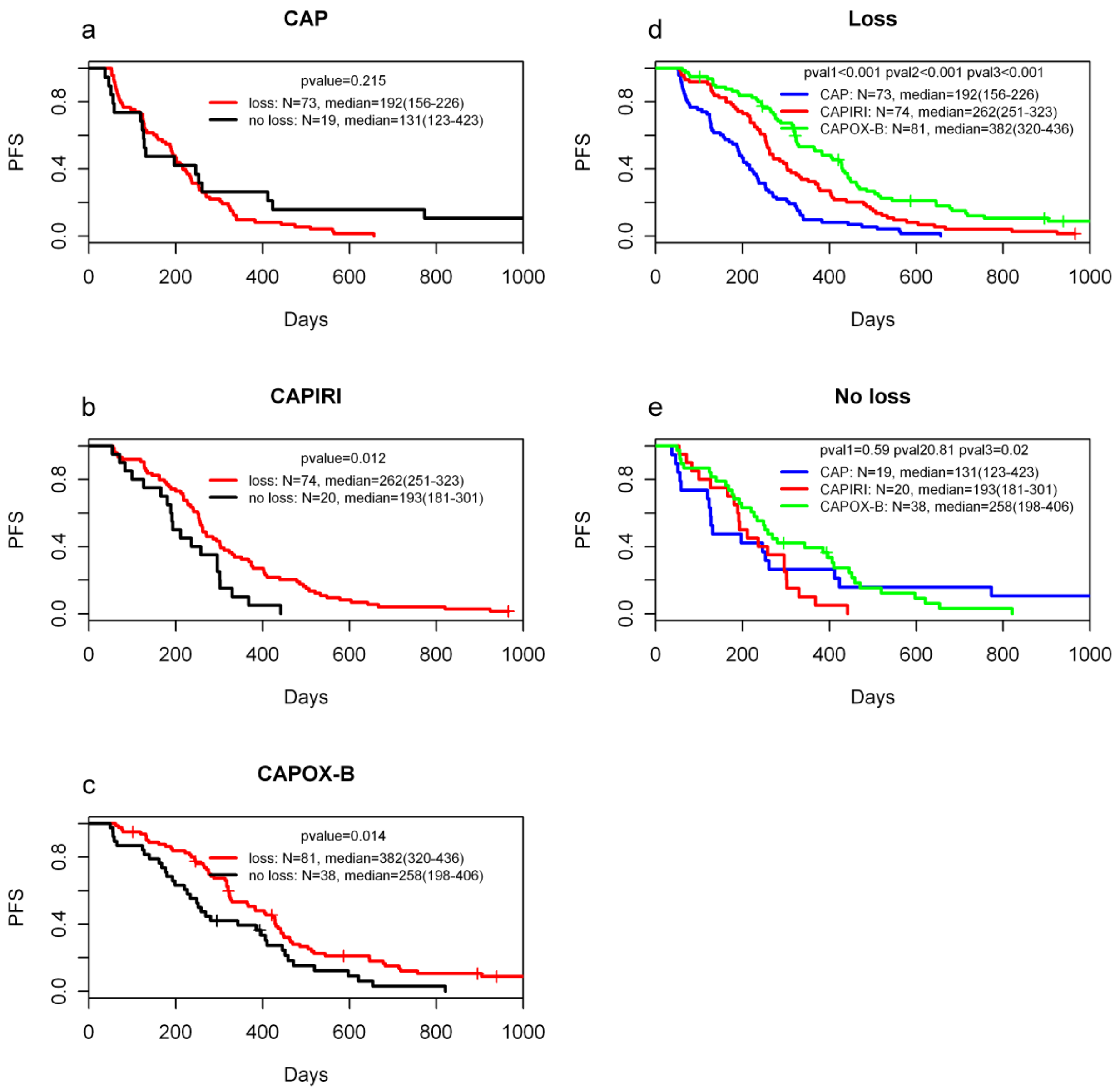

Supplementary Figure 8: Kaplan–Meier PFS analysis for chromosomal sub-region 18q11.2-12.1

Within patients groups a) CAP; b) CAPIRI; c) CAPOX-B, with loss in red, without loss in black. Between patient groups d) loss of 18q11.2-12.1; e) no loss of 18q11.2-12.1, with CAP in blue, CAPIRI in red, CAPOX-B green. Log-rank p-values, $p<0.05$ are considered significant; pval1, CAP versus CAPIRI; pval2, CAP versus CAPOX-B; pval3, CAPIRI versus CAPOX-B; N, number of patients; median, median survival with confidence limits within brackets and NA, no applicable value.

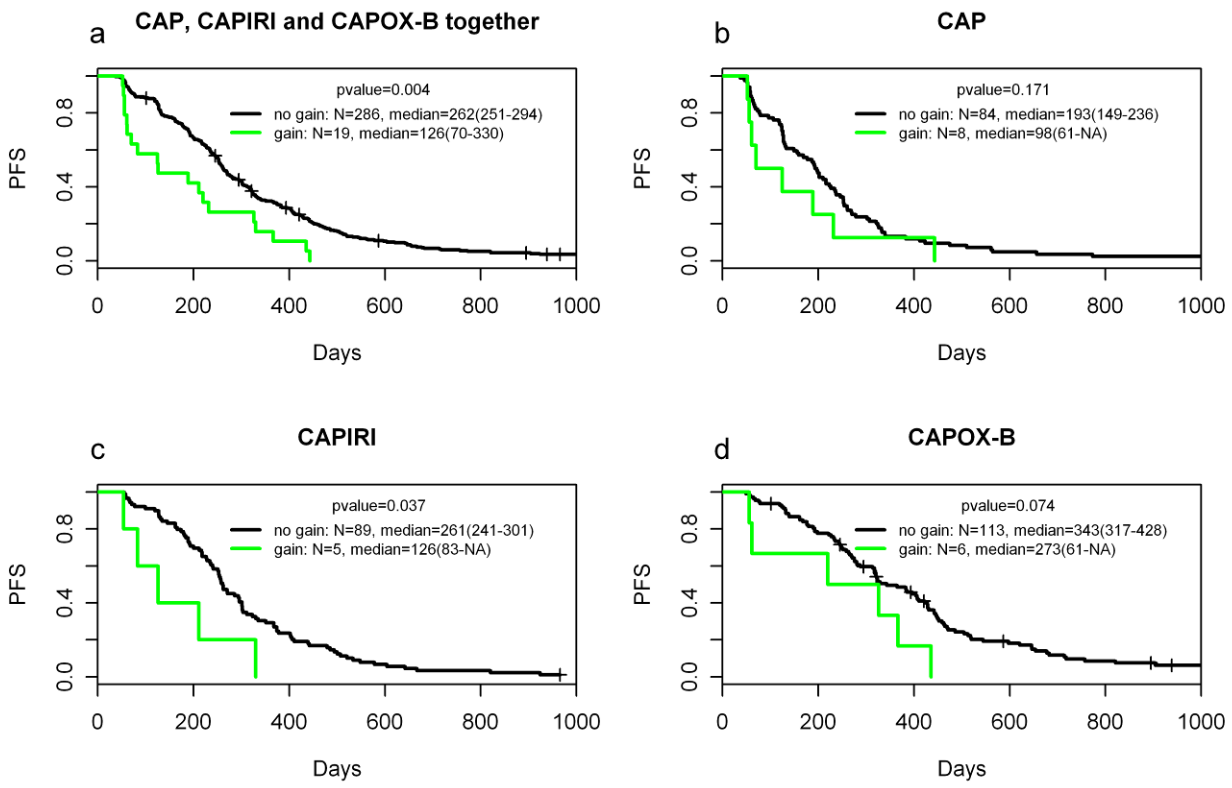

Supplementary Figure 9: Kaplan–Meier PFS analysis for chromosomal sub-region 9q12-q13

a) Three patients groups together; Within patients groups b) CAP; c) CAPIRI; d) CAPOX-B, with gain in green, without gain in black. Log-rank p-values, $p<0.005$ are considered significant; N, number of patients; median, median survival with confidence limits within brackets and NA, no applicable value.

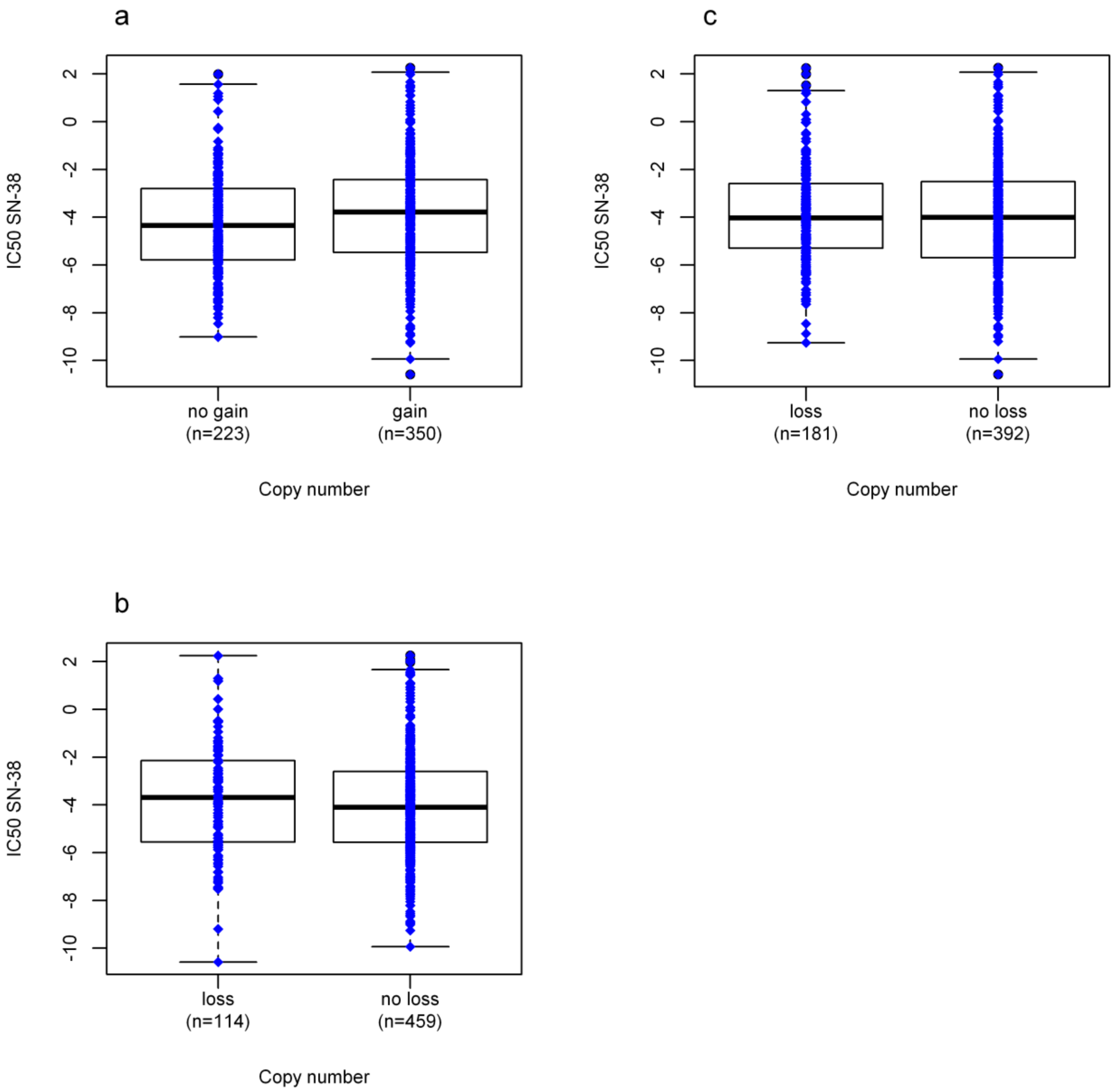

Supplementary Figure 10: SN-38 drug response for 573 cell lines for chromosomal regions 6q16.1−q21, 5q11.2-q13.2 and 18q21.1-q22.3

a) Box plot of 6q16.1−q21 copy number, uncorrected Kruskal-Wallis test p-value of 0.01; x-axis, gain (n=350) or no gain (n=223); y-axis, drug response (IC50-values). The error bars (whiskers) extend to the most extreme data point, but no further than 1.5 times the interquartile range. b) Box plot of 5q11.2-q13.2 copy number, uncorrected Kruskal-Wallis test p-value of 0.14; x-axis, loss (n=114) or no loss (n=459); y-axis, drug response (IC50-values). The error bars (whiskers) extend to the most extreme data point, but no further than 1.5 times the interquartile range. c) Box plot of 18q21.1-q22.3copy number, uncorrected Kruskal-Wallis test p-value of 0.45; x-axis, loss (n=181) or no loss (n=392); y-axis, drug response (IC50-values). The error bars (whiskers) extend to the most extreme data point, but no further than 1.5 times the interquartile range.

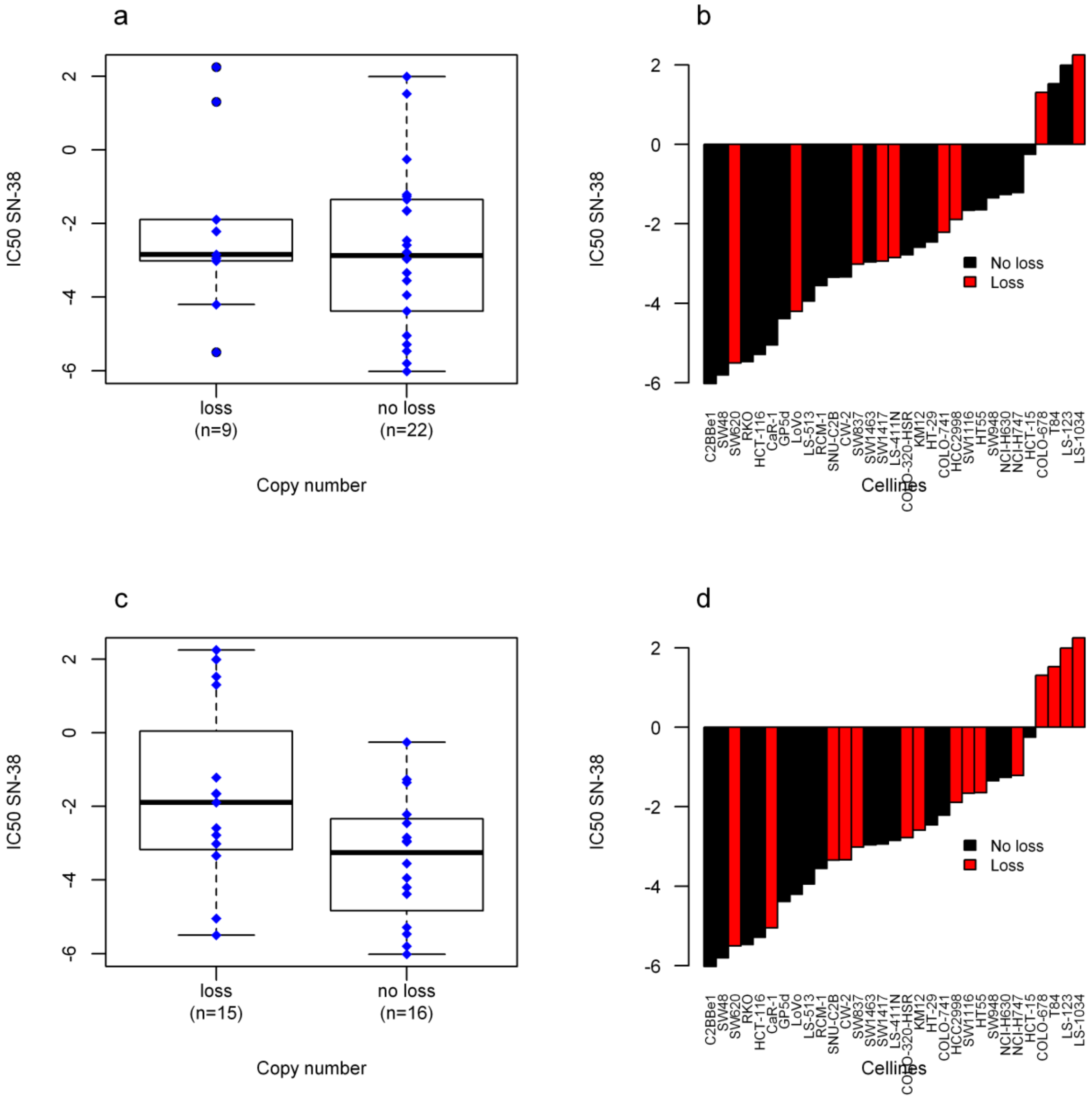

Supplementary Figure 11: SN-38 drug response for 31 CRC cell lines for chromosomal regions 5q11.2-q13.2 and 18q21.1-q22.3

a) Box plot of 5q11.2-q13.2 copy number, uncorrected Kruskal-Wallis test p-value of 0.63; x-axis, loss (n=9) or no loss (n=22); y-axis, drug response (IC50-values). The error bars (whiskers) extend to the most extreme data point, but no further than 1.5 times the interquartile range. b) Waterfall plot of 5q11.2-q13.2 copy number; x-axis, cell-lines ordered from left to right, for low to high IC50 value; y-axis, drug response (IC50-values); no loss, black; loss, red. c) Box plot of 18q21.1-q22.3copy number, uncorrected Kruskal-Wallis test p-value of 0.05; x-axis, loss (n=15) or no loss (n=16); y-axis, drug response (IC50-values). The error bars (whiskers) extend to the most extreme data point, but no further than 1.5 times the interquartile range. d) Waterfall plot of 18q21.1-q22.3 copy number; x-axis, cell-lines ordered from left to right, for low to high IC50 value; y-axis, drug response (IC50-values); no loss, black; loss, red.

Supplementary Table 1.

	**CAP (CAIRO) (N = 105)**	**CAPIRI (CAIRO) (N = 111)**	**CAPOX-B (CAIRO2) (N = 133)**
**Age**			
Age at randomisation	65 (40-83)	64 (35-81)	63 (36-83)
> 70 years	22%	26%	18%
**Gender**			
Male	63 (60%)	71 (64%)	73 (55%)
Female	42 (40%)	40 (36%)	60 (45%)
**WHOPerformance status**[#]			
PS0	69 (66%)	75 (68%)	86 (65%)
PS1	32 (30%)	29 (26%)	47 (35%)
PS2	4 (4%)	7 (6%)	
**Predominant localisation of metastases**[#]			
Liver	74 (70%)	79 (71%)	96 (72%)
Liver-only	45 (43%)	40 (36%)	45 (34%)
Extrahepatic	29 (28%)	31 (28%)	36 (27%)
Unknown	2 (2%)	1 (1%)	1 (0.7%)
**Number of affected organs**[*]			
1	57 (54%)	55 (50%)	58 (44%)
>1	48 (46%)	56 (50%)	75 (56%)
**LDH at randomisation**[#*]			
Normal	73 (70%)	80 (72%)	79 (59%)
Above normal	32 (30%)	31 (28%)	54 (41%)
**Prior adjuvant therapy**[#*]			
Yes	18 (17%)	19 (17%)	22 (17%)
No	87 (83%)	92 (83%)	111 (83%)
**Site of primary tumor**			
Colon	77 (73%)	66 (59%)	58 (44%)
Rectosigmoid	5 (5%)	8 (7%)	37 (28%)
Rectum	22 (21%)	36 (32%)	38 (29%)
Multiple tumors	1 (1%)	1 (1%)	

Supplementary Table 1. Baseline characteristics of the patients in the treatment groups CAP, CAPIRI and CAPOX-B.
[#]stratification factor CAIRO, [*] stratification factor CAIRO2.

Supplementary Table 2

region	band	CNA	chr	Kbstart	Kbend	treatment	nCNA	nNoCNA	pval PFS	Median PFS CNA	Median PFS noCNA	Genes CNA~mRNA expression
260	6q16.1-q16.3	Gain	6	95514	100707	CAPIRI	7	87	**0.002**	189(67-NA)	262(67-NA)	*KIAA0776 C6orf66 C6orf167 FBXL4 SFRS18 CCNC*
						CAP	6	86	0.74	116(58-NA)	192(133-232)	
						CAPOX-B	9	110	0.696	373(224-NA)	335(323-430)	
261	6q16.3-q21	Gain	6	100727	110015	CAPIRI	7	87	**0.002**	189(67-NA)	262(67-NA)	*ASCC3 ATG5 QRSL1 6orf203 PDSS2 LACE1 CD164 SMPD2 ZBTB24*
						CAP	6	86	0.74	116(58-NA)	192(133-232)	
						CAPOX-B	10	109	0.813	323(224-NA)	335(319-430)	
189	5q11.2	Loss	5	50533	51478	CAP	12	80	**0.003**	106(65-NA)	200(177-237)	
						CAPIRI	13	81	0.327	270(179-NA)	257(179-NA)	
						CAPOX-B	8	111	0.829	217(133-NA)	371(323-435)	
195	5q11.2-q12.1	Loss	5	56243	59531	CAP	17	75	**0.002**	106(65-133)	210(187-252)	*GPBP1**
						CAPIRI	16	78	0.723	302(200-410)	254(200-410)	
						CAPOX-B	17	102	0.168	253(196-417)	389(326-438)	
196	5q12.1	Loss	5	59548	59884	CAP	18	74	**0.002**	112(65-192)	210(187-252)	
						CAPIRI	18	76	0.953	286(200-403)	256(200-403)	
						CAPOX-B	19	100	0.179	253(196-479)	389(326-438)	
197	5q12.1	Loss	5	59911	62402	CAP	17	75	**0.001**	106(65-133)	210(187-252)	*DEPDC1B ELOVL7 KIF2A DIMT1L*
						CAPIRI	16	78	0.723	302(200-410)	254(200-410)	
						CAPOX-B	18	101	0.518	323(239-479)	389(319-436)	
198	5q12.1-q12.3	Loss	5	62417	66277	CAP	20	72	**<0.001**	106(65-189)	210(187-253)	*SFRS12IP1 SDCCAG10 CENPK PPWD1 SFRS12*
						CAPIRI	16	78	0.723	302(200-410)	254(200-410)	
						CAPOX-B	16	103	0.564	298(235-NA)	389(323-436)	
199	5q12.3-q13.1	Loss	5	66292	66528	CAP	23	69	**0.004**	123(67-192)	210(187-252)	
						CAPIRI	20	74	0.972	270(200-403)	254(200-403)	
						CAPOX-B	22	97	0.32	319(239-472)	390(326-436)	
200	5q13.1-q13.2	Loss	5	66544	68869	CAP	24	68	**0.004**	124(77-192)	202(177-253)	*SLC30A5 CCNB1 CENPH* MRPS36 CDK7 CCDC125 TAF9 RAD17*
						CAPIRI	20	74	0.972	270(200-403)	254(200-403)	
						CAPOX-B	22	97	0.188	274(239-373)	392(326-438)	
714	18q21.1-q21.33	Loss	18	45915	58546	CAPIRI	81	13	**0.002**	270(253-311)	181(253-311)	*MYO5B MBD1 CXXC1 C18orf24 ME2 ELAC1 SMAD4* MEX3C MBD2 POLI RAB27B CCDC68 TXNL1 WDR7 FECH NARS ATP8B1 ALPK2 MALT1 SEC11C LMAN1 PMAIP1 RNF152 PIGN KIAA1468* TNFRSF11A ZCCHC2 PHLPP*
						CAP	77	15	0.072	190(133-226)	197(119-773)	
						CAPOX-B	99	20	0.408	335(323-435)	256(179-528)	

region	band	CNA	chr	Kbstart	Kbend	treatment	nCNA	nNoCNA	pval PFS	Median PFS CNA	Median PFS noCNA	Genes CNA~mRNA expression
715	18q21.33	Loss	18	58560	58966	CAPIRI	80	14	**0.002**	271(254-311)	186(254-311)	*PHLPP BCL2*
						CAP	78	14	0.184	187(129-223)	217(131-773)	
						CAPOX-B	99	20	0.398	335(323-435)	256(179-528)	
716	18q21.33	Loss	18	58968	59137	CAPIRI	81	13	**0.002**	270(253-311)	181(253-311)	*BCL2*
						CAP	81	11	0.162	187(129-223)	237(193-NA)	
						CAPOX-B	101	18	0.536	335(323-430)	302(179-631)	
717	18q21.33	Loss	18	59150	59397	CAPIRI	80	14	**0.002**	270(254-311)	186(254-311)	*KDSR VPS4B*
						CAP	77	15	0.105	189(129-226)	197(127-773)	
						CAPOX-B	100	19	0.396	335(323-435)	256(179-631)	
718	18q21.33-q22.3	Loss	18	59414	70375	CAPIRI	78	16	**0.001**	270(254-323)	192(254-323)	*SERPINB8 TMX3 RTTN SOCS6* C18orf55 CNDP2*
						CAP	75	17	0.092	189(129-226)	197(127-423)	
						CAPOX-B	97	22	0.202	371(323-436)	238(170-466)	
217	5q31.1-q31.3	Loss	5	135034	139486	CAPOX-B	14	105	**0.002**	237(170-430)	390(326-438)	*BRD8 KIF20A CDC23 ETF1 HSPA9 CTNNA1* SIL1 UBE2D2*
						CAP	19	73	0.242	127(106-278)	199(169-234)	
						CAPIRI	12	82	0.864	270(200-NA)	256(200-NA)	
218	5q31.3-q32	Loss	5	139498	146912	CAPOX-B	14	105	**0.002**	237(170-430)	390(326-438)	*C5orf32 PFDN1 ANKHD1SLC35A4 IK* WDR55 HARS HARS2 DIAPH1 HDAC3 KIAA0141 RNF14 NDFIP1 LARS RBM27 TCERG1*
						CAP	15	77	0.78	127(118-333)	193(149-232)	
						CAPIRI	12	82	0.782	270(200-NA)	256(200-NA)	
220	5q33.1	Loss	5	148726	149538	CAPOX-B	20	99	**0.003**	196(133-335)	392(326-443)	*PCYOX1L CSNK1A1*
						CAP	16	76	0.287	127(118-278)	193(156-232)	
						CAPIRI	16	78	0.757	298(200-439)	254(200-439)	
222	5q33.2-q34	Loss	5	154550	162780	CAPOX-B	14	105	**0.001**	215(133-430)	390(326-438)	*MED7 THG1L CLINT1 RNF145 UBLCP1 TTC1 SLU7 PTTG1*
						CAP	13	79	0.29	127(106-NA)	193(149-232)	
						CAPIRI	12	82	0.782	270(200-NA)	256(200-NA)	
223	5q34	Loss	5	162801	162867	CAPOX-B	15	104	**<0.001**	196(133-335)	390(326-443)	*CCNG1*
						CAP	19	73	0.204	177(126-269)	197(149-232)	
						CAPIRI	13	81	0.849	270(200-NA)	256(200-NA)	
224	5q34-q35.3	Loss	5	162876	180154	CAPOX-B	16	103	**0.001**	237(133-430)	390(326-438)	*RARS CCDC99 NPM1 ERGIC1 C5orf25 HSPC111 HIGD2A FAF2 GPRIN1 UIMC1 LMAN2 GRK6 DDX41 RMND5B HNRNPAB CLK4 ZNF354A ZNF354B RUFY1 CANX MAML1 MAPK9 CNOT6*
						CAP	11	81	0.206	126(106-NA)	197(156-234)	
						CAPIRI	11	83	0.241	311(241-NA)	256(241-NA)	
673	17q12	Loss	17	34144	35068	CAPOX-B	16	103	**0.004**	278(235-414)	389(323-443)	*PSMB3 PIP4K2B CCDC49 RPL23 LASP1 RPL19 FBXL20 MED1 CRKRS NEUROD2 STARD3*
						CAP	14	78	0.774	209(127-510)	189(131-232)	
						CAPIRI	14	80	0.496	226(189-820)	258(189-820)	

region	band	CNA	chr	Kbstart	Kbend	treatment	nCNA	nNoCNA	pval PFS	Median PFS CNA	Median PFS noCNA	Genes CNA~mRNA expression
676	17q21.2	Loss	17	35826	36961	CAPOX-B	16	103	**0.004**	278(235-414)	389(323-443)	*TOP2A SMARCE1 TMEM99 KRTAP3-3 KRTAP1-1*
						CAP	14	78	0.774	209(127-510)	189(131-232)	
						CAPIRI	15	79	0.63	218(179-521)	261(179-521)	
677	17q21.2-q21.31	Loss	17	36980	40902	CAPOX-B	16	103	**0.004**	278(235-414)	389(323-443)	*EIF1 NT5C3L KLHL11 ACLY NKIRAS2 KAT2A COASY MLX EZH1 VPS25 CCDC56 BECN1 PSME3 RUNDC1 RPL27 BRCA1 NBR2 NBR1 DUSP3 TMEM101 LSM12 TMUB2 GPATCH8 CCDC43 EFTUD2 NMT1 MAP3K14*
						CAP	14	78	0.911	176(118-510)	190(133-232)	
						CAPIRI	15	79	0.63	218(179-521)	261(179-521)	
703	18q11.2	Loss	18	17834	17906	CAPOX-B	78	41	**0.004**	390(329-450)	256(201-403)	
						CAP	71	21	0.148	192(149-226)	189(123-423)	
						CAPIRI	68	26	0.065	262(240-335)	244(240-335)	

Supplementary Table 2. Chromosomal region significantly associated with PFS (p-value <0.005)

Sub-regions in rows and concatenated total regions, alternatingly shaded with a grey or white background. Region, sub-region number; band, chromosomal band; CNA, copy number aberration: gain or loss; chr, chromosome; Kbstart, start in Kb; Kbend, end base positions; treatment, treatment group; nCNA, number of patients with the copy number aberration; nNoCNA, number of patients without the copy number aberration; pval PFS, p-value progression-free survival; Median PFS CNA, median PFS with the copy number aberration; Median PFS noCNA, median PFS without the copy number aberration; Genes CNA~mRNA expression, the genes located on this region of which a correlation of copy number with mRNA expression was found. * mutated in data from the “The genomic landscapes of human breast and colorectal cancers” manuscript[1]); [#] mutated in data from capecitabine toxicogenomics pathway regions [2-6]

Supplementary Table 3

region	band	CNA	chr	Kbstart	Kbend	treatment	nCNA	nNoCNA	p-value	Median PFS CNA	Median PFS noCNA	Genes CNA~mRNA expression
185	5p14.3-p13.3	Gain	5	18703	34133	CAPIRI	23	71	**0.047**	241(179-303)	262(179-303)	*RNASEN C5orf22 GOLPH3 MTMR12 ZFR SUB1 TARS*
						CAP	9	83	0.85	202(125-NA)	189(131-232)	
						CAPOX-B	18	101	0.468	323(179-553)	371(323-436)	
185	5p14.3-p13.3	Loss	5	18703	34133	CAP	9	83	**0.007**	106(62-NA)	199(169-236)	
						CAPIRI	3	91	NA	368(301-NA)	256(301-NA)	
						CAPOX-B	4	115	NA	217(69-NA)	371(323-435)	
830	Xp22.33	Gain	23	1782	2451	CAPIRI	31	63	**0.009**	335(218-485)	254(218-485)	
						CAP	22	70	0.924	197(156-261)	182(126-226)	
						CAPOX-B	32	87	0.823	328(274-453)	371(292-436)	
830	Xp22.33	Loss	23	1782	2451	CAP	8	84	**0.01**	66(61-NA)	197(159-236)	
						CAPIRI	3	91	NA	71(67-NA)	258(67-NA)	
						CAPOX-B	8	111	0.283	307(239-NA)	349(323-430)	
698	18p11.32	Loss	18	112	1792	CAPIRI	64	30	**0.04**	262(241-330)	236(241-330)	*USP14 THOC1 C18orf56 TYMS*# *ENOSF1 YES1*
						CAPOX-B	74	45	0.031	389(328-449)	263(201-413)	
						CAP	67	25	0.465	193(156-236)	131(123-261)	
699	18p11.32-p11.21	Loss	18	1798	11639	CAPIRI	66	28	**0.05**	262(241-330)	211(241-330)	*METTL4 NDC80 SMCHD1 EMILIN2 LPIN2 MRCL3 MRLC2 ZFP161 RAB12 KIAA0802 NDUFV2 ANKRD12 TWSG1 RALBP1 PPP4R1 VAPA NAPG*
						CAPOX-B	75	44	**0.048**	389(328-443)	268(224-417)	
						CAP	68	24	0.377	192(149-236)	160(123-261)	
700	18p11.21	Loss	18	11655	13997	CAPIRI	67	27	**0.05**	262(251-311)	211(251-311)	*CHMP1B MPPE1 IMPA2 TUBB6 AFG3L2 CEP76 PSMG2 PTPN2 SEH1L CEP192 C18orf19 RNMT*
						CAPOX-B	75	44	**0.039**	389(328-443)	268(224-417)	
						CAP	66	26	0.411	190(133-236)	191(123-261)	
705	18q11.2	Loss	18	19434	19807	CAPIRI	74	20	**0.012**	262(251-323)	193(251-323)	*LAMA3**
						CAPOX-B	81	38	**0.014**	389(326-443)	263(201-413)	
						CAP	73	19	0.215	192(156-226)	131(123-423)	
707	18q12.1	Loss	18	23552	25278	CAPIRI	79	15	**0.015**	262(251-323)	193(251-323)	
						CAPOX-B	89	30	**0.036**	373(326-443)	256(196-417)	
						CAP	74	18	0.208	192(156-232)	129(119-423)	

Supplementary Table 3: Chromosomal sub-regions significantly associated with PFS in two treatment groups (p-value <0.05).
Chromosomal region significantly associated with PFS (p-value <0.005). Sub-regions in rows and concatenated total regions, alternatingly shaded with a grey or white background. Region, sub-region number; band, chromosomal band; CNA, copy number aberration : gain or loss; chr, chromosome; Kbstart, start base position in Kb; Kbend, end base positions in Kb; treatment, treatment group; nCNA, number of patients with the copy number aberration; nNoCNA, number of patients without the copy number aberration; p-value PFS, p-value progression-free survival; Median PFS CNA, median PFS with without the copy number aberration; Median PFS noCNA, median PFS without the copy number aberration; Genes CNA~mRNA expression, the genes located on this region of which a correlation of copy number with mRNA expression was found. * mutated in data from the "The genomic landscapes of human breast and colorectal cancers" manuscript[1]); # mutated in data from capecitabine toxicogenomics pathway regions [2-6]

Supplementary Table 4

Band	CNA	chr	Kbstart	Kbend	p-value
9q12-q13	gain	9	66462	70092	0.004
1p31.1	loss	1	83392	83496	0.005
5q23.1-q31.1	loss	5	120130	131740	0.004
5q31.1-31.3	loss	5	135034	139487	0.003
5q33.2-q43	loss	5	154550	162780	0.005
5q34	loss	5	162801	162867	0.000

Supplementary Table 4: Chromosomal sub-regions significantly associated with PFS in the three treatment groups together (p-value <0.005)

Band, chromosomal band; CNA, copy number aberration: gain or loss; chr, chromosome; Kbstart, start base position in Kb; Kbend, end base positions in Kb; p-value, p-value progression-free survival (PFS).

Supplementary Table 5

Band	Sub-regions	CNA	pvalue all cancer cellines	pvalue only CRC cellines
5q11.2-q13.2	189,195-200	Loss	0.13	0.63
6q16.1-q21	260-261	Gain	0.01	0.05
18q21.1-q22.3	714-718	Loss	0.45	0.05

Supplementary Table 5: Chromosomal sub-regions associated with SN-38 drug response

Regions per row. Band, chromosomal band; Sub-regions, sub-regions in the total region; CNA, copy number aberration: gain or loss; p-value all cancer cell lines, p-value IC50 all 573 cancer cell lines; p-value only CRC cell lines, p-value IC50 31 CRC cell lines.

Supplementary Table 6

GeneID	Chr	Frequency	Drug
SGK3	8	1	
MAPK15	8	3	
ADCK5	8	3	
IKBKB	8	4	
RIPK2	8	1	
*FGFR1**	8	8	pazopanib
DYRK4	12	3	
*FLT3**	13	5	CHIR-258, tandutinib, sorafenib, lestaurtinib, CGP 41251
*FLT1**	13	4	sunitinib, pazopanib, axitinib, CEP 7055
*BC048278**	13	3	sunitinib, pazopanib, axitinib, CEP 7055
*CDK8**	13	1	flavopiridol
DGKH	13	1	
NEK5	13	1	
NEK3	13	1	
TP53RK	20	1	
SGK2	20	4	
STK4	20	3	
PTK6	20	5	
SRMS	20	5	
UCKL1	20	5	
URKL1	20	5	
HCK	20	1	
PCK1	20	2	
MYLK2	20	1	
C20orf111	20	1	

Supplementary Table 6: Kinases and their frequencies of amplifications.

Genes are ordered by chromosome. GeneID, gene name of the kinase; Chr, chromosome; Frequency, number of patients with the amplified gene; Drug, drug by which the gene is targeted. *known drug target in IP.

Supplementary Table 7

Gene ID	Chr	Frequency	Ligands	Drug
*FGFR1**	8	8	*FGF1, FGF2, FGF3, FGF4, FGF5, FGF6, FGF9, FGF10, FGF11, FGF12, FGF12B, FGF13, FGF14, FGF16, FGF17, FGF18, FGF19*	pazopanib
*FLT3**	13	5	*FLT3LG*	CHIR-258, tandutinib, sorafenib, lestaurtinib, CGP 41251
*FLT1**	13	4	*VEGFA, VEGFB, PGF*	sunitinib, pazopanib, axitinib, CEP 7055
TNFRSF6B	20	5	*TNFSF6, TNFSF14*	

Supplementary Table 7: Receptors and their frequencies of amplifications.

Genes are ordered by chromosome. GeneID, gene name of the receptor; Chr, chromosome; Frequency, number of patients with the amplified gene; Drug, drug by which the gene is targeted. *known drug target in IPA.

Supplementary Table 8

Gene ID	Chr	Frequency	Receptors	Drug
*VEGFA**	6	2	*FLT1, KDR, NRP1, NRP2*	bevacizumab, ranibizumab, aflibercept, pegaptanib
FGF6	12	3	*FGFR1, FGFR2, FGFR4*	
TNFSF11	13	1	*TNFRSF11A, TNFRSF11B*	
FGF9	13	1	*FGFR1, FGFR2, FGFR3, FGFR4*	
BMP7	20	2	*BMPR1A, BMPR1B, BMPR2, ACVR1, ACVR2, ACVR2B*	

Supplementary Table 8: Ligands and their frequencies of amplifications.

Genes are ordered by chromosome. GeneID, gene name of the ligand; Chr, chromosome; Frequency, number of patients with the amplified gene; Drug, drug by which the gene is targeted. *known drug target in IPA.

Supplementary Table 9

Gene	Frequency
MYC	5
AKT2	5
ERBB2	3
KRAS	2
CAMK2B	2
STAT1	2
STAT4	2
PLCG1	1
NRG1	1
EGFR	1
ERBB4	1
MAP3K1	1
PIK3CA	1
SP1	1
STAT3	1
STAT5A	1
STAT5B	1

Supplementary Table 9: EGFR pathway and their frequencies of amplifications

Genes are ordered by frequency. Gene, gene name; Frequency, number of patients with the amplified gene. Genes not listed had a frequency of 0.

Supplementary References

1. Wood,L.D. *et al*. The genomic landscapes of human breast and colorectal cancers. *Science* **318,** 1108-1113 (2007).

2. Yang,W. *et al*. Genomics of Drug Sensitivity in Cancer (GDSC): a resource for therapeutic biomarker discovery in cancer cells. *Nucleic Acids Res* **41,** D955-D961 (2013).

3. Whirl-Carrillo,M. *et al.* Pharmacogenomics knowledge for personalized medicine. *Clin. Pharmacol. Ther.* **92,** 414-417 (2012).

4. Thorn,C.F. *et al.* PharmGKB summary: fluoropyrimidine pathways. *Pharmacogenet. Genomics* **21,** 237-242 (2011).

5. Marsh,S. *et al.* Platinum pathway. *Pharmacogenet. Genomics* **19,** 563-564 (2009).

6. Maitland,M.L. *et al.* Vascular endothelial growth factor pathway. *Pharmacogenet. Genomics* **20,** 346-349 (2010).
